# What is Basic Research? Insights from Historical Semantics

**DOI:** 10.1007/s11024-014-9255-0

**Published:** 2014-06-24

**Authors:** Désirée Schauz

**Affiliations:** Technische Universität München, Fachgebiet Technikgeschichte, c/o Deutsches Museum, Museumsinsel 1, 80538 Munich, Germany

**Keywords:** Basic research, Applied research, Pure science, Applied science, Historical semantics, Science policy, History of science, Germany, United States of America, 19th century, 20th century, Uncertainty

## Abstract

For some years now, the concept of basic research has been under attack. Yet although the significance of the concept is in doubt, basic research continues to be used as an analytical category in science studies. But what exactly is basic research? What is the difference between basic and applied research? This article seeks to answer these questions by applying historical semantics. I argue that the concept of basic research did not arise out of the tradition of pure science. On the contrary, this new concept emerged in the late 19th and early 20th centuries, a time when scientists were being confronted with rising expectations regarding the societal utility of science. Scientists used the concept in order to try to bridge the gap between the promise of utility and the uncertainty of scientific endeavour. Only after 1945, when United States science policy shaped the notion of basic research, did the concept revert to the older ideals of pure science. This revival of the purity discourse was caused by the specific historical situation in the US at that time: the need to reform federal research policy after the Second World War, the new dimension of ethical dilemmas in science and technology during the atomic era, and the tense political climate during the Cold War.

For some years now, the concept of basic research has been under attack. Its relevance has been questioned empirically as a result of changes in academic research, normatively with respect to science policy, and even theoretically in science and technology studies. Yet while the significance of the concept is in doubt, basic research is still a very common analytical category, deployed not least as a means of distinguishing the new future science policy from the old ideal of basic research. But what exactly is basic research? What is the difference between basic and applied research? Aside from a few exceptional studies (Calvert 2006; Godin 2005a; Pielke 2012), science studies have only just begun to seriously reflect upon these questions. When and why did the concept of basic research emerge in the first place? Is the ideal of basic research nothing more than a relaunch of the older pure-science discourse? Historical semantics appears to be a useful approach for answering these questions because its historical perspective provides the conceptual clarity required both in current debates in science and technology studies and public debates on science policy.

In the 1990s, sociological studies claimed that science was undergoing profound changes. Since then, prominent labels such as “Mode 2” or “triple helix” have come to signify a new way of organizing science and technology that transgresses institutional boundaries between universities, industry, and governmental research. According to the alleged paradigm shift from Mode 1 to Mode 2, application-oriented research programmes with cooperative and transdisciplinary project teams have replaced the former university-centred basic research mode. Proponents of this new way of comprehending knowledge production even call for science policy to be modified in order to reflect the altered research mode (Gibbons et al. 1994; Etzkowitz and Leydesdorff 1997). Our “Leonardo world”, as portrayed by Jürgen Mittelstraß, is ruled by the imperative of technology. The interplay of science and technology raises society’s expectations of research applications, even when the outcomes sometimes turn out to be risky (Mittelstraß 1994). These arguments have certainly shaped the debates in science and technology studies and science policy in recent years, although discussions about the degree of change and how to evaluate it remain controversial (Weingart 2008; Greenberg 2007).

According to studies addressing these historical shifts in science, basic research determined the status quo ante. These studies describe basic research as an application-disinterested mode of research embedded in a disciplinary and academic setting that contrasts, in respect of every analytical feature, to Mode 2. The concept of Mode 1, however, is not based upon profound historical analysis; it rather appears to represent the previously prevailing sociological perspective on science in the tradition of Robert Merton, who emphasized disinterestedness and universalism as central characteristics of modern science. Yet historical studies suggest that the way in which science was organized had already undergone significant change in the early 20th century, as politicians, scientists, and industry formed a new alliance from which all three groups hoped to benefit (Ash 2002; Mowery and Rosenberg 1993).

Moreover, although recent debates in science studies have demonstrated high levels of discontent with the notion of basic research, producing instead new analytic labels like triple helix or Mode 2, the term “basic research” and its antonym “applied research” continue to frame the discourse about science, without any awareness of both terms’ historical conditionality as discursive strategies in research policy. The semantic dichotomy merely gives way to a continuum between basic and applied research in which the favourite mode, the “use-inspired basic research” (in German “*anwendungsorientierte Grundlagenforschung*”), is located somewhere in the middle of the continuum (Stokes 1997; Mittelstraß 1994). However, aside from the motif of application, we lack an explicit set of distinctive criteria because studies persist in assuming basic research to be a given category.

In other studies, categories such as basic and applied research no longer play a major role. Research grounded in approaches such as actor-network theory, that is studies emphasizing the societal context of science, anthropological studies focusing on day-to-day laboratory work, and the new – although still vague – concept of technoscience are united in their critique of discursive boundaries, which they claim to obstruct the view on the reality of research. While research dealing with Mode 2 indicates the change within the historical development of science, those supporting these new approaches call for a change in theoretical perspective. Bruno Latour, one of the most famous proponents of this idea, identifies demarcations such as nature/society or science/technology as a typically modern delusion covering, albeit quite successfully, the hybrid character of research (Latour 1993). Claiming an overall paradigm shift for the social sciences, Latour suspects that traditional sociology has frozen thought within boundaries and institutional separations in its studies for quite a long time and levels his criticisms at a static display of society blind to the dynamics of interactions (Latour 2005). For Latour, the distinction between basic and applied research is supposed to be part of these delusive demarcations: such a simple dichotomous order cannot represent the “complicated and unpredictable relations between scientists and other agencies” (Latour 1987: 117). Latour argues that the high esteem in which basic science is held does not correspond with the reality of technoscience. In his early call for the concept of technoscience, he even argued statistically, interpreting the high proportion of spending on development and applied research in contrast to that spent on basic research evident in research and development statistics as indicative of the real importance of technology and the level of overall support it receives within society (Latour 1987: esp. 168–173).

Latour’s argument about modern delusions and his opposition to a basic-research-centred perspective on science have found resonance among some historians of science. For instance, Peter Dear identifies the ideology of modern science as misrepresenting the reality of research in the natural sciences. Although, according to Dear, some effort has been made to integrate the instrumental and useful character of the natural sciences in the tradition of science since Francis Bacon, natural philosophy, with its ideal of contemplative understanding, has retained the upper hand (Dear 2005: 404). From an historical point of view, this discrepancy between the philosophical notion of science and research practice appears as an anachronism requiring explanation. In general, criticism levelled at the long-prevailing ideal of pure science has led to a reorientation in the history of science that includes the applied side of science and opens up the field to the history of technology (Forman 2010). Recent studies look beyond the academic core – the universities – and into industrial laboratories, where the majority of researchers have worked throughout the 20th century (Shapin 2008).

Despite this growing awareness of the ideological or normative character of basic research, the majority of historians still use the concept as a given, analytical category without questioning its relationship to varying historical contexts. Studies on German war-time science, for instance, try to determine to which extreme of the basic-applied continuum the examined research projects tended.^1^ As to the history of US science and innovation policy, the concept of basic research seems to be inevitably associated with the name of Vannevar Bush and the reorganization of US science after the Second World War. The basic-applied taxonomy is therefore primarily regarded as representation of the institutional logic of modern research organization: the so-called linear model which coined the idea of innovation process for so many years. Investigating the negotiation of science policy in the 1940s, historical studies have revealed dissenting political preferences and conflictive institutional interests, demonstrating that the post-war order in science policy had initially been highly contested. However, although the historical contingency of the concept has thus become more and more apparent, many historians still do not reflect on the meanings and functions of the concept of basic research. Even the meteoric career of this relatively young term does not seem to be puzzling historians. They rather interpret the concept as additional part of an existing taxonomy, “supplementing” the former “language of pure and applied science” (Dennis 2004: 225). As a result, the concept of basic research has been locked up in a black box next to “pure science” whose meaning is also still enigmatic (Galison 2008). It is only recently that the investigation of shifting functions, varying meanings and symbolic dimensions of the concept of basic research – beyond the institutional level of research organization and funding – have become an object of interest in the history of science (Krige 2006).^2^

What remains of the current debates in science and technology studies is the question as to why demarcations such as basic and applied research have occurred at all. If Latour is right in pointing out that the concepts of basic and applied research do not represent actual research practices, why have these terms become so important? Which (other) functions have they fulfilled? More precisely, what has “basic research” meant for the identity of science and for its relationship to technology? Which role has the concept of basic research played in science policy, that is in the negotiations between science and society about aims and values of research? And how has the concept affected the public image of science?

This article therefore seeks to analyze the genesis of the concept of basic research up until the early 1960s, by which time it had become a common concept in science policy in the West. It will also take a brief look at discourses on pure science prevalent in the 19th century as a means of establishing the effects of historical legacy and variation over time. This study has two central aims. Firstly, it intends to detect the different semantic dimensions of basic research – its institutional, epistemic, ethical, social, and political attributions. Secondly, it discusses the significance of the concept of basic research in the natural sciences, in research policy, and in science studies: to which historical challenges faced by research in the 20th century did the concept of basic research respond?

I argue that the concepts of basic research and fundamental research did not arise out of the 19th-century tradition of pure science, which had idealized research as an intrinsically philosophical search for eternal truth. On the contrary, these new concepts emerged in the late 19th and early 20th centuries at a time when society’s expectations regarding the utility of science were rising sharply. In the knowledge that research output is hard to predict, scientists used these concepts to bridge the gap between the promise of utility and the uncertainty of scientific endeavour. Only after 1945, when US policy strongly shaped the notion of basic research, did these concepts revert to the older ideals of pure science. In order to understand this revival of the purity discourse, we need to take the specific historical situation of the post-war US into account, in particular the new plans for federal funding of research, the new dimension of ethical dilemmas faced by science and technology following Hiroshima, and the overall political climate of the Cold-War era. The insights gained from historical semantics show that basic research was not – and cannot be – considered a clearly distinguishable analytical mode of research. After 1945, the concept of basic research formed part of a discursive strategy that adjusted scientific research to complex and even contradictory societal requirements; it was for these socio-political reasons that the concept became so important. Consequently, moral and ideological attributions were and still are inseparably tied to the concept of basic research.

American and German discourses provide the empirical basis of this study. Yet this article is not intended as a fully-fledged comparative study of two countries. Rather, I analyze Germany and the US because these countries were considered best-practice models in science at varying points in time and they both share a long history of mutual exchange and learning. At different points in time, each of the two countries allows us to trace the emergence and evolution of specific understandings of the role of science in society. The first section on the older pure-science ideals of the 19th century revolves mainly around Germany, which had become a leading science nation at that time. In the following section, which discusses how the concept of basic research emerged and evolved until 1945, the German experience also takes centre stage. The third section covers US science policy from the Second World War until the early 1960s, when the term basic research had become established as a key concept in science policy. The article ends, on a more comparative note, with a short history of the concept of basic research in post-war Germany. The second and the third sections overlap in time because the Second World War and the post-war period require a more comparative perspective. For a long time, scientific research during the Nazi period was thought to represent a turning away from all fundamental principles of science. The war, however, confronted both US and German scientists with similar political demands and requirements. After 1945, US policy became a role model for the Federal Republic of Germany (West Germany). Before the empirical analysis commences, however, the next section will introduce readers to historical semantics and discuss how I will use this approach to structure the empirical discussion.

## Some Remarks on Historical Semantics

This study resorts to approaches in conceptual history and discourse analysis. Discourse analysis fits with the research questions for several reasons. Firstly, it is designed to make visible what is taken for granted when people think or talk about social phenomena and the implicit rules that apply in the practice of framing topics. Secondly, discourse analysis identifies classifications and demarcations, such as the distinction between basic and applied research, as essential strategies in discursive practice. Thirdly, it is based on the assumption that discursive production is historically contingent. Whereas discourse analysis strives, in the main, to analyze patterns of assertions, conceptual history focuses on semantics and key concepts. Especially the latter takes the polysemy of language and communication into account. Moreover, conceptual history’s foundation in the philosophy of history means that it offers us assumptions about semantic shifts over time.

In contrast to the tradition of semantic analyses in the philosophy of science, which is mainly interested in the epistemic impact of metaphors (Blumenberg 2010), my study is based on a strand of historical semantics rooted in historical studies on the dawn of modernity. It focuses on key concepts in social and political language. If we assume that basic research is largely a concept of science policy or of negotiations between the scientific community and the public, then this approach seems more suitable for this study. Moreover, conceptual history is embedded in reflections about the philosophy of history. According to Reinhart Koselleck, the major proponent of the German school of conceptual history, a shifting societal dictionary – the emergence of neologisms or changes in semantic attributions – indicates historical upheaval. Key concepts and parts of their meanings, however, may persist, so that old and new semantic dimensions coexist. Koselleck’s approach thus corresponds with approaches in the philosophy of history that take different layers of time into account. Koselleck clearly demonstrates that language is not an epiphenomenon of reality, but rather that it frames both human experience and the way in which society perceives the world. He conceives key concepts as cognitive strategies designed to deal with reality, especially in situations where expectation and experience diverge. Ideologies, in particular, are supposed to compensate semantically for a lack of convergence between expectations and experiences (Koselleck 2006: 85).

Whereas Koselleck’s conceptual history defines key concepts primarily as cognitive strategies of the human that deal with reality, discourse analysis goes further in assuming that discursive strategies might serve various societal functions. In his commentary on the concept of the dispositive, Michel Foucault emphasized that discourses, non-discursive practices, institutions, and objects are linked by common strategic functions. This does not mean, however, that the outcome of such a strategic dispositive necessarily corresponds to the initial function. On the one hand, novel discourses have the power to set new practices or different forms of institutional organization. On the other hand, it is also possible that emerging discourses provide existing institutions or operations with new legitimacy. The history of dispositives also turns out to be quite complex. Taking Foucault’s remarks on the philosophy of history into account, the concept of the dispositive is quite similar to Koselleck’s idea of a complex history of different layers of time lying upon one another (Schauz 2010).

Since discourse analysis has progressed by adapting aspects of polysemy, the combination with conceptual historical approaches has become more obvious. One approach appears to be particularly fruitful for investigating the history of basic research: Jürgen Link’s idea of “collective symbols”, which came about when Link dealt with the problem of interdiscursive processes. Link believes that multiple meanings of metaphors and symbols are capable of linking different discourses demonstrating diverse patterns of assertions (Link 1986). In other words, metaphors can bridge discursive gaps. With regard to this study, science policy may be described as one such interdiscursive process in which scientific expectations encounter society’s expectations. And, without anticipating the detailed analysis of the concept of basic research below, it is obvious that “basic” as the first part of the compound offers a variety of possible interpretations.

Of course, discourse analysis also has a tradition in science studies, in particular regarding demarcation discourses. Most relevant in this context is Thomas F. Gieryn’s study (1999) on the cultural boundaries of science, which he identifies as resulting from professional boundary work. According to Gieryn, boundary work does not represent fixed or institutional demarcations, but is rather a dynamic process of negotiations with contested boarders and regenerated situations of uncertainty. Gieryn stresses that boundaries linked to key concepts such as pure science vary according to special situations and social circumstances. Unlike Gieryn, however, I do not expect that discursive practices revolving around basic research are strategies exclusively used by scientists to protect their professional interests. Moreover, I doubt that the discursive function of basic research can be restricted to boundary work.

In summary, this study is based upon the followings assumptions derived from conceptual history, discourse analysis, and studies on scientific boundary work. The attributions and linked demarcations of basic research are expected to vary according to space and time. Prior semantic dimensions, however, might persist or experience revival. The emergence of basic research as a new term may at least indicate an historical shift in either science or its role in society. The abstractness of the term basic research offers a wide range of meanings and discursive strategies. The concept has the potential to function as a collective symbol for science policy that links different discourses within society. Given its variability, this key concept of science policy, together with its antonyms, cannot be interpreted as representing fixed institutional boundaries. Rather, the concepts seem to emerge in situations of uncertainty or cognitive dissonance. Yet they may legitimize the institutional organization of research or define operative goals. Moreover, the discourses revolving around basic research communicate a wide range of ideals, expectations, promises, as well on professional and public claims.

Finally, there are some preliminary methodological remarks that need to be addressed. Although the study focuses on the concept of basic research, it also has to detect conceptual variations and alternative or concurrent terms, not to mention antonyms. Relevant terms for the US case are basic research, fundamental research, pure science and basic science. Antonyms and concurrent terms like applied research, applied science, contract research and mission-oriented research are included as far as they are needed to analyze the meanings of basic research, but their own conceptual histories will not be analyzed at full length. For the German case, these terms are *Grundlagenforschung*, *reine Wissenschaft*, *reine Forschung*, *angewandte Forschung*, *angewandte Wissenschaft* and *Zweckforschung*.

With regard to conducting the discourse analysis, it was most relevant to compile a broad sample of documents enabling me to identify prevalent, repeated patterns of assertions.^3^ Besides key texts from scientists well-established in research organization, the sample also covers texts produced for normal-science communication.^4^ The study is thus based on published documents relating to science policy as well as on scientific articles and books. Especially the volumes of the American journal *Science* and its German counterpart *Die Naturwissenschaften* have been subjected to systematic analysis. Furthermore, electronic search functions, in particular those enabling full-text searches with the keywords listed above, have been most useful for periods in which concepts were not yet commonplace. The digital library of Google Books is an important tool for historical semantics because it enables us to detect texts which might otherwise be overlooked by more traditional research strategies based on library holdings and cross references. As such, Google Books provides a unique tool for tracing both the emergence and diffusion of concepts. However, given that text acquisition in Google Books is dynamic and not entirely transparent to the user, it is difficult to delineate the corpus of books actually contained within its database. Thus Google Books may not be easy to use for scholars interested in exact bibliometric analysis, but it can help researchers gain a rough idea of when certain concepts began to be used and how use of these concepts became more or less common across different periods of time and within different language communities. This is how the current article uses the information derived from Google Books.

## Pure Science in the 19th Century: The Natural Sciences and the Philosophical Tradition of Academia

As studies have so far located the concept of basic research in the tradition of pure-science ideals, the following section will deal with the term’s prehistory as a means of tracking continuities and breaks in the way science perceived itself. The notion of pure science and the conceptual opposition between “pure” and “applied” in science can be traced back to the 18th century. The attributes of “pure” and “applied” referred in turn to the much older, classical distinction between theory and practice that had undergone reinterpretation during the Scientific Revolution. Back then, Francis Bacon and his contemporaries had tried to conflate the new empirical and instrumental form of knowledge of nature with the older tradition of natural philosophy and its idea of contemplative understanding (Dear 2005: 393–397). In the late 18th century, these attributes became important once again for natural scientists positioning themselves within the academic community for the purposes of finding a way into the university system. Although states such as Prussia demanded ever more instrumental knowledge and technical education for their mining industries or other state-owned enterprises (Klein 2010), natural scientists had to adjust to the predominant philosophical understanding of science^5^ at universities, which, even then, consisted only of philosophical, theological, legal, and medical faculties.

In the case of chemistry, Christoph Meinel has already demonstrated that, in the Age of Enlightenment, chemists labelled their discipline as “pure and applied” so that chemistry could become an acceptable subject at universities, shedding its older status as an auxiliary science of medicine (Meinel 1985). Due to its empirical approach and its utilitarian orientation, chemistry was still classified as an “art” rather than as a “science” in the 18th century. Academic teaching had hitherto focused on imparting theoretical knowledge and established theorems, that is pure science. In contrast, the applied sciences represented experience-based knowledge on the epistemic level; at the same time “applied” denoted research with a practical purpose. Both aspects of these so-called applied sciences did not (yet) fit into the philosophical tradition of universities. By striving to become a part of this academic institution, chemists had to stress both the pure scientific and applied aspects of their discipline (Meinel 1985; Bud and Roberts 1984).

At the very same time, philosophy was engaged in reviving the controversy between rationalism and empiricism that solidified a hierarchical concept of knowledge. As a consequence of the philosophical longing for the wholeness and absoluteness of ideas, *a posteriori* approaches continually played a subordinate role in contrast to *a priori* and metaphysical ways of knowing (Ross 1962: 68–69). The concept of cognition process in science turned out to be one-way: from the general to the particular. This concept of scientific progress implied the possibility of deducing endless applications and specific, context-linked knowledge from universal principles such as the laws of nature. The advancement of knowledge, however, was not supposed to take place the other way around. This distinction between pure and applied science thus corresponded to institutional and epistemic settings in the scientific community of the late 18th and 19th centuries.

### The Natural Sciences Face Challenges from Engineering and Technological Success

In the mid-19th century the pure/applied boundary started focusing on the distinction between the natural sciences and technology. The common definition of technology as applied natural sciences represented a special version of this one-way concept of knowledge. This definition was widespread – even economists believed in the one-way relationship between science and technology. They assumed that only scientific discoveries and theories paved the way for innovations: “Technical science may stimulate pure science to a certain extent, but, on the whole, technology is much more at the receiving end. Pure science is always further ahead of applied science, and never the other way round. However, technology finally turns science into a common good” (Rössler 1857: 179, translation by DS).

It was above all the community of natural scientists that wanted to preserve the hierarchical distinction between science and technology. The scientific foundation and the aspiring academic status of engineering in the second half of the 19th century challenged the scientific profession, in particular physicists (Gieryn 1999: 51–62). As the natural sciences had only recently assumed their place within the university, the legacy of natural philosophy and its epistemic and moral ideals, such as the unrewarded dedication to science for its own sake, was even stronger than the century before (Dear 2005: 401–404). Having scarcely ascended to the league of the pure sciences, the natural sciences even adopted the idea of an eternal truth defined by the discovery of natural laws.

The words of German physician Rudolf Virchow represent this adapted concept of pure science, but, more importantly, they also show that this purity discourse was not without contradictions. With the economic success of technical innovations and the growing appreciation of engineers within society throughout the German Empire, Virchow and his colleagues increasingly forged a link between themselves and the promise of technical progress in order to promote the idea of indispensable scientific endeavour:

All the benefits that have emerged from the steam engine, from telegraphy, photography, chemical discoveries, the production of colours and so on and so forth, all these benefits are based on scientific theorems that we men of science have unveiled, and not until we are absolutely sure that they are laws of nature, we pass these truths on to the general public so that others can work with them and create new things that nobody could imagine before, that no one has ever dreamt of, that see the light of day for the first time and transform the character of society and the state. (Virchow 1877: 8–9, translation by DS)

Compared with the great engineering inventions of the 19th century and their noticeable effects on everyday life and society as a whole, scientific progress was less visible. In a way, this poor visibility was one aspect of the ideal of the pure scientist in its philosophical tradition: a scholar who, in solitude, dedicates life and work to science, driven by the sole motive of finding the truth – or at least contributing his tiny part to the scientific community’s joint effort – even without any prospect of public acknowledgement. In fact, as Peter Dear put it, “the authority of science in the modern world rests to a considerable extent on the idea that it is powerful, that it can do things” (Dear 2005: 404). Yet, the scientific strategy of technological promise in order to gain greater visibility, support, and acknowledgment appeared risky; the scientific pledge to technological progress needed a show of confidence. Given the uncertainty and contingency of scientific advancement, it seemed even harder to predict if or when discoveries would lead to new technologies. Scientists thus defined their work as a long-term endeavour in contrast to engineering, which they classified as a medium-term project aimed at satisfying immediate need. In any case, the fact that researchers such as the chemist Justus von Liebig felt it necessary to defend the scientific profession reflects the growing pressure the scientific community faced from societal expectations in the course of the 19th century:

Even the most powerful effect of science on the life and spirit of men is so slow, noiseless, creeping and barely perceptible that a superficial observer would be hard pressed to assess its impact. The expert, however, knows that no real progress in this world is currently achieved without science and that the accusation whereby it is not of public benefit preoccupies the general public and not the men of science, who each in their own way, unwaveringly follow their goals. Indeed, they remain untroubled about the future benefits of their work since these accrue neither to them nor to an individual country but to the whole of mankind. (Liebig 1862: 33, translation by DS)

### Blurring Boundaries in the Late 19th and Early 20th Centuries: Scientists in Transition

The fact that scientists felt compelled to do boundary work indicates that scientific practice had already begun to change and that the hierarchical epistemic order no longer applied across the board. It was the birth of engineering as an academic discipline that set off this dynamic process of boundary work. By acquiring the right to award doctorates in the late 19th and early 20th centuries, the German technical colleges enhanced their academic status (König 1999). Leading figures of this new group of aspiring engineers such as Alois Riedler, a mechanical engineer and rector of the Technische Universität Berlin-Charlottenburg from 1899 to 1900, persistently stressed that the relationship between science and technology was a two-way process:

Technology has its natural share in the progress of the natural sciences; in many areas technology has even run ahead of the natural sciences until deeper scientific insights in turn paved the way for perfecting technical development; … [T]hrough the magnificence of its tangible achievements, technology has raised the public’s awareness of the natural sciences and has contributed enormously to making science, in general, more popular. (Riedler 1900: 12, translation by DS)

Conversely, scientists themselves began to overcome the gap between (pure) science and technology. Related distinctions, for instance, between discovery and invention were also blurring. Within the expanding field of the natural sciences in the late 19th century, researchers had to transcend the limits of both established disciplines and methods in order to find out something new. The development of instruments became, more than ever before, an integral part of scientific work; the act of designing new techniques became as relevant as discovering new elements or laws of nature. The instrumentality of science, not only in terms of its methodological role of confirming theories but also in terms of its effectiveness, had finally become part of the image of the truthfulness of science in the modern world (Wilhelm Ostwald 1929: 21; Dear 2005: 404; Joerges and Shinn 2001).

Scientists such as the Nobel Prize winner and pioneer of physical chemistry Wilhelm Ostwald campaigned for closer cooperation between scientists and engineers. While criticizing the old supremacy of natural philosophy, he emphasized the similarities of scientific and technological endeavour, in particular a systematic approach to research and to the desire to venture into the unknown (Wilhelm Ostwald 1908: 20). As far as Ostwald was concerned, scientists and engineers nonetheless differed in terms of their motivations (or goals) and their temporal perspective; having discovered a new technology, engineers abandoned scientific questioning, whereas scientists followed the path to its very end, hoping to find definitive explanations to their questions. Although this notion of the advancement of knowledge was less asymmetric than it had been a few decades earlier, the emphasis Ostwald placed on science’s long-term orientation and the continued ideal of human curiosity as a scientific value in itself demonstrated that a sense of the moral superiority of science endured. (Wilhelm Ostwald 1905, 1911).

While the ideals of pure science were in the process of dissolving, by 1900, both the institutional settings of research and research practices in the natural sciences had already undergone significant change. The emergence of professional industrial laboratories with salaried researchers (initially in the chemical and electrical industry), the establishment of special research institutes outside of the universities (both national laboratories in the service of the state and research centres for specific research fields with mixed funding), the beginning of special funding programmes for science, and the more extensive involvement of the administration in science policy issues were some of the developments in science and in the attitudes within society towards science observable in different countries.

Studies into German science emphasize that two new types of institutes, the *Notgemeinschaft der deutschen Wissenschaft* (Emergency Association of German Science) and the *Kaiser-Wilhelm-Gesellschaft* (Kaiser Wilhelm Society), concluded an ongoing process of change in science at an institutional level that had come about in response to the limitations of the former university-centred organization of research and to the new expectations of industrialized mass society (Szöllösi-Janze 2005; Ash 2002: 35–38).^6^ The Kaiser Wilhelm Society, established in 1911 to promote the natural sciences in Germany, was a reaction to the increased requirements of disciplines such as chemistry and physics as well as a response to increasing industrial demand for scientific knowledge and growing international competition. With the financial support of both the state and influential entrepreneurs, scientists in the institutes on material research belonging to the Kaiser Wilhelm Society were able to concentrate their entire efforts on research, that is “pure science”, without needing to undertake teaching duties. The Emergency Association of German Science largely sponsored research projects at the universities. This fund, derived from a variety of sources and governed by academics, had been initiated by scientists after the First World War.

The funding programme *Gemeinschaftsforschung* (Collaborative Research), which sought to further public health, the economy, and the greater public good, together with the research areas pursued by several institutes belonging to the Kaiser Wilhelm Society provide evidence that the pure-science ideal was becoming less important. These self-governed academic institutions promoted research that responded directly to industrial and political demands. Collaborative Research, for example, financed projects which promised to either secure the production of raw materials or develop substitute materials, to improve material processing or technological development, and to increase food production.

To sum up the whole section, the historical overview from the 19th to the early 20th century shows that the pure-science ideal prevailed until the late 19th century when the cooperation between university scientists and industry started to become closer. The pure-science ideal was a legacy of the long-standing domination of philosophy in academic culture. Having worked hard to earn the status of academic disciplines, it was difficult for the natural sciences to overturn a notion of science that strove for eternal truth while ignoring the technical and economic fruitfulness of research. The fact that natural scientists continued to cling to the philosophical tradition, however, became a point of conflict in the late nineteenth century because the high social esteem enjoyed by the natural sciences was based primarily on their significance for technological innovation and economic success. German science had already begun to adjust to the new role of science in society on an institutional level, the conceptual distinctions between pure and applied science and between science and technology were set to blur in the early 20th century.

## Science in the First Half of the 20th Century: Fundamental Research and the Promise of Utility

The scientific purity discourses lost importance around 1900 and new terms began to reshape the notion of science. This semantic shift suggests that the role of science in society had already changed. The German composite noun *Grundlagenforschung* (fundamental research),^7^ is a relatively young term that first emerged in the early 20th century within a very specific context in the discipline of mathematics (Dingler 1911: 35; Rulf 1913). In the late 19th century, mathematics underwent a disciplinary realignment known as mathematical modernism (Mehrtens 1990). German mathematicians played a leading role in this scientific movement, the main goal of which was disciplinary autonomy. The movement’s proponents created a special, self-referential language by freeing the discipline from any metaphysical grounds and providing mathematics with a theoretical framework that denied any reference to reality or other concepts in science and technology and favoured instead an intrinsic, formal logic. Journal articles such as “Mathematische Probleme” by David Hilbert (1901) delineated a future research programme for mathematics revolving around principal epistemic questions of proof. In summary, modern mathematicians created a new epistemic foundation for their discipline.

Although the role of applied mathematics was an issue for dispute within this reform movement, the term fundamental research was not actually used as an antonym that contrasted to applied mathematics. Within the particular context of mathematics, fundamental research denoted studies that contributed to solving fundamental logical problems like those Hilbert had put on the agenda. Herbert Mehrtens (1990: 149) thus classifies fundamental research as a specific subdiscipline (“*Spezialdisziplin*”) within mathematics. Because this specific meaning was confined to mathematics, the term fundamental research first spread to adjacent disciplines such as philosophy and, in particular, the philosophy of science (Lewin 1922). In fact, the German version of fundamental research was not common throughout the 1920s and early 1930s, and the few times the term emerged, it referred mostly to fundamental epistemic questions within disciplines.

In contrast to the German scientific discourse, the English term “fundamental research” emerged slightly earlier and, more importantly, within a different context than in Germany. The English term basic research was initially less prevalent. Roger Pielke has detected a *New York Times* article from 1919 in which “basic research” emerged in the context of a Congressional hearing on agricultural policy. According to him, the concept was an offspring of the political discourse since its use was restricted to the political arena until the late 1930s (Pielke 2012: 343). It must be added that “fundamental” and “basic” were, among other things, used as attributes to denote the core academic disciplines, such as physics, mathematics, or chemistry, upon which other disciplines were founded. Thus, fundamental science and basic science meant something completely different to fundamental research or basic research in the English/American context.

The initial use of fundamental research in fields such as plant breeding and technological or industrial research indicates that the term did not emerge from the 19th-century purity discourse. In the 1890s, scientists of agronomy at the American land-grant colleges called for more fundamental research in general aspects of plant physiology in order to continue making progress in plant breeding (Arthur 1895: 360). Problem- and application-oriented research led them to new questions that “pure” botany had not yet raised. The land-grant colleges were the result of a federal initiative to foster education in agronomy and technology, and to offer higher education to the wider public. As a result of their agricultural focus, these colleges were provided with federally controlled land to establish agricultural experiment stations. Similar to the German technical colleges, the land-grant colleges were not originally on an equal footing with the universities in terms of scientific prestige (Thelin 2004: 135–137). Yet researchers in these experimental centres faced high public expectations to provide results that could improve farming practices and increase crop yields (Marcus 1985).

The demand for more fundamental research expounded one problem: the uncertainty of scientific outcomes, even if a project had a clear task to fulfil right from the start. Given this uncertainty, doing fundamental research meant at least promising to lay a cornerstone for future technologies, new products, or new materials. If research failed to produce new knowledge proving useful, scientists could still legitimise their work via the ideal of pure science, that is the advancement of knowledge as a value in itself. As any reference to the intrinsic ideal of pure science was secondary, it served primarily as a back-up means of legitimisation and only secondarily as a way to claim recognition for applied botany among “pure” scientists. In the end, similar to the German example in engineering, scientists in applied botany declared the distinction between pure and applied science to be invalid: “All science is one. Pure science is often immensely practical, applied science is often very pure science, and between the two there is no dividing line” (Coulter 1917: 228). Applied botanists called upon science to remain open to everyday needs and problems (Coulter 1919: 366). Alongside these examples from botany, the term fundamental research can be found very early on in the context of technological and industrial research. Fundamental research denoted any scientific research revolving around basic technical problems with the goal of improving existing technology or, hopefully, developing new technology (Nutting 1917: 250).

The fact that the concept of fundamental research arose in research fields with an explicit application-orientation reveals that the new term was not a synonym for pure science. Rather, it conveyed the promise that science would produce, sooner or later, useful knowledge. This semantic shift was a response to the growing expectations of science within society and the increasing number of possibilities that scientific research had been able to offer in the development of technology and other societal improvements since the late 19th century. However, researchers and scientists phrased their promise of utility very cautiously; the metaphorical meanings of “fundamental” express the idea that research is the first, but not the only step in a complex process. Hence, the strategic use of the term can be described as twofold: to promise utility and, at the same time, to confine expectations that may be far too high.

With respect to British science policy in the first half of the 20th century, Sabine Clarke (2010) has already pointed out that fundamental research did not emerge as a synonym for pure science. She shows that in Britain, the new Department of Scientific and Industrial Research, established in 1916, used the term first and foremost to stimulate industrial research. The new ministry was supposed to coordinate and support research that promised economic and social improvement. At first, manufacturers and scientists scarcely welcomed the new grants offered by the Department; according to Clarke, both parties wanted to avoid any kind of governmental interference. Confronted by this industrial opposition, the Department of Scientific and Industrial Research advertised long-term research projects dealing with the basic properties of materials or with technical processes with the new term “fundamental research”. In this particular context, the label pure science would have evoked the image of curiosity-driven research without any practical end.

As Clarke demonstrates, the new term can only be understood within its specific institutional and national setting; thus, we should not be too rash to conclude that the findings of the British study also apply to the German case. Furthermore, Robert Kline’s older study (1995) on the boundary discourse of pure and applied science in the US, which focuses on engineering and its relationship to the natural sciences, suggests that, even in the English-speaking world, the meaning of the term fundamental research varied greatly. According to Kline, the distinction between “pure” and “applied” had only become common in the 1870s, and so the ideal of pure science was a relatively recent phenomenon in the US. Although the demarcation between pure and applied science was becoming blurred in the interwar period, Kline argues that the majority of researchers in engineering eventually adopted the pure-science ideal in order to underscore their scientific capabilities and their growing professional status. Kline’s main argument is that because engineering was unable to assert an autonomous ideal of itself, technological knowledge continued to be subordinated to scientific knowledge in the 20th century. For Kline the new term fundamental research represented a modified ideal of pure science which could also apply to technology. Where engineering is concerned, Kline admits that he is unable to identify a clear strategy of autonomy forming an essential aspect of the traditional notion of pure science.

### Nazi Opposition to the Notion of Pure Science

In Germany, the term *Grundlagenforschung* only became common in the sciences during the late 1930s. Its meanings certainly deviated from the original use of the concept within the context of German mathematics, as well as from the old semantics of pure science. After the scientific purity discourse ran out of steam in the 1920s, the National Socialist German University Lecturers’ League (*Nationalsozialistischer Deutscher Dozentenbund*), which represented the younger generation of lecturers attempting to bring the universities into line with Nazi ideology in particular, fought against the institutional, epistemic, and normative concepts that characterised the ideals of pure science (Nagel 2008). The Nazi discourse denounced the 19th-century humanistic notion of academia as a liberal bourgeois ideal that had permanently estranged science and scholarship from the German people.

On a detailed scale, the Nazi discourse criticized the older concept of science as being a selfish project pursued by scientists. This criticism was levelled at the epistemic norm of objective neutrality and the assumption that the natural sciences were unconditional – in particular in terms of the choice of research subjects – thus exposing the notion of pure science as a concept contrived by the ivory tower. Furthermore, Nazi critics blamed the self-referential concept of pure science for causing institutional fragmentation and disciplinary differentiation in science. Continuing the Weimar policy of collaborative research, the Nazi scientific ideal entailed joint efforts by researchers from different institutional and disciplinary backgrounds aimed at solving the problems of the day; problems that were, of course, defined by the politics of the Nazi regime. It is no surprise that the Nazi counter-concept of science quite openly called for a politicization of the academic world – in particular with regard to staff and research policy – and reinterpreted the ideals of universalism, academic freedom, and unity of science in light of the *Volksgemeinschaft* ideology (the ideology of the community of German people): academic universalism transformed into social universalism, which sought to overcome individual, institutional, and disciplinary interests. The political interpretation of freedom meant that science was in a position to contribute to the German people’s independence from foreign raw materials, in accordance with the Nazi quest for autarky. And lastly, by invoking the older ideal of the unity of science, they legitimized collaborative science, its different disciplines, and its various institutions in order to fulfil national tasks (Henkel 1933; Krieck 1933; Löhr 1938; W. Schultze 1938).

### Research in Nazi Germany: Between Four-Year Plans and Long-Term Science Policy

In light of the official campaign against the old pure-science ideal at the beginning of the Nazi regime, the use of fundamental research in the late 1930s can hardly be understood as a new version of pure science presenting the search for knowledge of nature and truth both as an a priori goal of research and a value in itself. The terms *Grundlagenforschung* and *Zweckforschung* (goal-oriented research) gained hold as political efforts to acquire control over academic and industrial research increased. In 1937, the Nazi regime established a research council, the *Reichsforschungsrat* (Reich Research Council), which was responsible for funding research. During the war, the Research Council was directly responsible to the Army Ordnance Office (Flachowsky 2008: 232–462).

The Research Council’s first president, military general and professor of army technology Karl Becker, defined fundamental research as science that could not be “commanded and accelerated”. He guaranteed, therefore, that “as far as researchers and facilities in the institutions [for fundamental research] in question offer even some guarantee of success”, there would be no interference from the Research Council (Becker 1937: 26). Becker made particular mention of the various institutions for aeronautical research and the institutes of the Kaiser Wilhelm Society, promising to abstain from exerting any control over these institutions in light of their close relationships to industry. Goal-oriented research, which was meant to be built on fundamental research, was to fit into the schedule of the four-year plan. In this context, goal-oriented research denoted first and foremost industrial research leading to the development of advanced technology. Against the backdrop of the four-year plan, the Nazi regime demanded that industry give complete insight into its research activities (Becker 1937: 25, 27).

In 1940, the *Illustrierte Zeitung*, a well-established illustrated magazine published in Leipzig, devoted an entire issue to the topic of German research in the service of the people in order to present Nazi science policy. The magazine included articles from leading scientists such as the biochemist and Noble Prize winner Adolf Butenandt, journalists specializing in scientific topics such as Hans Hartmann^8^, and ministry officials (No. 4956, 22 August 1940). To some extent, the issue was a response to continuing foreign criticism of the way the Nazis had incorporated German academia into National Socialism (Rust 1940; Hartmann 1940). Completely ignoring criticism of racist staffing policy, the articles presented a concept of science that responded to the needs of society without compromising scientists’ research freedom. “The freedom of research would not be endangered when the state ensures that state-funded institutes are given the task of conducting fundamental research in order to solve problems within the national economy” (Krauch 1940: 122, translated by DS).

The articles, however, also addressed German scientists on the question of how a more utility-oriented research affected its institutional setting. The issue of organizing science in order to quickly achieve societal and technological progress without duplicating efforts in both academic and industrial research had already been under discussion within the paradigm of rationalisation prior to the Nazi’s seizure of power. From the late 19th century onwards, industry conducted more and more research in its own laboratories, and the good salaries attracted talented researchers. The future role of universities as training and research institutions and the initial division of labour between academic and industrial research thus became a vital question of science policy. Furthermore, the changing research practices also led to an organizational discussion about individual or team research. The terms fundamental research and goal-oriented research were part of these ongoing negotiations (Krauch 1941: 2; Brüche 1944: 114–115; Stadlinger 1944: 227, 229; Verein Deutscher Chemiker 1943; Drescher-Kaden 1941: 10, 16–17).

Overall, the articles in this special issue sought mainly to demonstrate to the public how German scientists, whose work was less visible, contributed to the nation during war time. Authors such as Butenandt tried to explain their ongoing experimental work in terms of both its meaning for society and its potential impact to a wider lay audience (Butenandt 1940). Following the initial hostility demonstrated by Nazi ideology towards the academic elite and elitist institutions such as the academies of sciences, this issue of the *Illustrierte Zeitung* promoted science wholesale by emphasizing that it was necessary for society to support research.

Within the natural sciences up to 1939, the new term “fundamental research” was rarely used and did not yet have an established, fixed set of meanings. In physics, for example, fundamental research could denote theoretical physics or, alternatively, it referred to the older distinction between the natural sciences and technology (Reichenbächer 1937: 285; Hiedemann 1939: V, 1). Despite this semantic variation, the strategic uses of the new term in most of the disciplines bore some resemblance to one another when it came down to combining the term with goal-oriented research. It is striking that as the term fundamental research became more widespread after 1939, scientists tended to mention *Grundlagenforschung* and *Zweckforschung* in the same breath (Witzell 1944: 212–217). In fact, the term fundamental research emerged in the natural and technical sciences mainly when the individual field of research was close to application or demonstrated promise for military, economic, and political aims. This was the case, for instance, with innovations in weaponry and military strategy, maintaining public health, ensuring food supply, rationalizing the production and use of raw materials, inventing substitute materials, and encouraging industrial production. In the humanities, the term fundamental research was still less common. This observation leads us to the question of whether the use of the two terms really worked as a boundary discourse.

Interdisciplinary research fields, such as forestry, represented a utility-oriented notion of science in the first place. In the case of forestry, research promised more profitable cultivation and effective technical treatment of the raw material wood. Germany’s rise as a colonial power in the late nineteenth century had already transformed forestry into a politically and economically significant discipline, fostered since by the German state. In the Nazi war economy, the issue of raw materials, and with it the supply of wood, gained even greater importance (Steinsiek 2008). In this disciplinary context, fundamental research and goal-oriented research represented two equivalent sub-areas of forestry: one that studied the nature of the substance wood, and one that analyzed its material properties and the effects of technical treatment. The overall goal of both research fields was to acquire knowledge about the optimal use for the raw material wood (Runkel 1942: 305–306).^9^

The majority of scientists defined fundamental research as pursuing fundamental questions of nature, its substances, and its processes. This contrast to goal-oriented research still adhered to the old demarcation between nature, on the one hand, and society and its relationship to natural resources, on the other. But questions about nature, labelled by scientists as fundamental research in the late 1930s and 1940s, arose within the context of technical and practical problems (Kaiser Wilhelm-Gesellschaft zur Förderung der Wissenschaften 1939: 322; Hoffmann and Suhr 1944: 550), that is in applied science disciplines such as aeronautics, armament, forestry, plant breeding, and nutrition.

In the majority of cases, scientists simply stressed the necessity of both fundamental research and goal-oriented research, in other words, the general necessity of research for any kind of progress. This is where the views of the scientific community converged with the goals of Nazi economic policymakers, who were aware that the US and British governments were providing massive support to research for economic and military purposes (Krauch 1939). When it came to clearly defining terminology in this period, scientists surprisingly described fundamental research as the study of nature, devoid of any concrete notion of how it might be applied in terms of technology or societal utility. Yet having just drawn a distinction between utility-oriented research and research driven simply by the urge for knowledge, scientists immediately strove to emphasize that limiting fundamental research was not possible in terms of research practice and its institutional settings, whether in industry, in universities, or in other research institutions (Bauermeister 1938; Wolfgang Ostwald 1942: 130–131; Niemeier 1944: 106–107). Moreover, the distinction between fundamental research and goal-oriented research was often criticized as misleading because it suggested that fundamental research was far removed from any notion of useful application (Zenneck 1944: 10; Endell 1942: 113; Wolfgang Ostwald 1942: 130–131).

These definitions must be seen as a vestige of patterns characterizing scientists’ former understanding of science. However, one question remains unanswered: if this differentiation of research types appeared to have little consequence for the scientific community, why did scientists introduce new terminology that could be understood as part of a dichotomy and that, moreover, was reminiscent of former boundary discourses? As the use of the term fundamental research was prevalent in engineering as well as in those research fields in chemistry, physics, biology, and geography that responded, in particular, to the concrete needs of the economy and the political regime, the intention was hardly to reactivate either the old demarcation between science and technology or the ideal of science for its own sake. The fact that new terms emerged reveals two things. Firstly, under the Nazi regime the scientific community felt the need to renegotiate the conditions under which science and research were conducted. Secondly, the old concepts of science no longer fit with existing practices in science.

The term fundamental research was fresh; *Zweckforschung*, which was highly unusual in the natural sciences until the mid 1930s, was even more so.^10^ In fact, the latter only gained importance during the Nazi period. Some scientists explicitly considered goal-oriented research as a temporary focus of science responding to a situation of national emergency. In 1936, the chemist Wolfgang Ostwald, son of Wilhelm Ostwald and former president of the *Kolloid-Gesellschaft* (Colloid Society), stated that “[o]ver the last years, much has been said about so-called ‘Zweckforschung’. It means the entirety of efforts to draw more extensively than usual on scientific research for solving major economic problems” (Kolloid-Gesellschaft 1936: 159, translation by DS). To be precise, the term goal-oriented research was spreading at the very same time that the creation of the new government administration in 1937, the Reich Research Council, institutionalised the four-year plan. This new authority and the second four-year plan that ensued broadened the field of activities in which science henceforth was understood as an important prerequisite for economic progress. Thus, in contrast to free research, goal-oriented research meant target-oriented research according to the goals of the four-year plan (Bachér 1937; Willing 1937).^11^

In order to explain the emergence of this new scientific nomenclature, it is most revealing to look at the chronology governing the spread of new terms. With the foundation of the new Reich Research Council (1937), which claimed to bring German research efficiently into line with Nazi policies, some scientists were concerned about the future funding of fundamental research (Bauermeister 1938: 476). It would be misleading to interpret this plea for fundamental research as a struggle for freedom of science that ignored the expectations of society in favour of absolute professional independence; the concerns expressed do not reveal an objection to the idea that science should serve political aims or national tasks. Yet the scientists’ worries certainly revolved around the question of how to govern science. The concern was, in fact, that scientific knowledge as a resource for innovation might dwindle in the long run. It can be described as an argument of knowledge sustainability meaning that knowledge will run short if scientists and policymakers align knowledge production exclusively with immediate needs. Within this sustainability discourse, fundamental research represented the experiences that, firstly, scientific progress was often unexpected and, secondly, that even the research output that sought practical solutions was unpredictable and needed time before its application was possible. According to these researchers, science had to conquer new ground deemed necessary for the long-term advancement of technology. Only a few scientists actually recognized the semantic shift in scientific nomenclature and criticized the new term fundamental research for constraining science to technological ends (Richter 1943: 207).

The reference to the long-term and unpredictable nature of scientific research was, of course, not new. Back in the 19th century, this had already served as an argument in the science-technology boundary discourse. Yet it was not until the 20th century that this aspect of scientific and technological progress became an everyday experience in many fields of research. The problem of how to find the right balance between venturing into the unknown and, at the same time, abiding by a research policy that sought to keep the aims of research in its sights had been under discussion in the 1920s, in particular within the context of industrial research. Faced by the Nazi Regime’s four-year plan and the increasing pressure of the expectations on science during the war, it became even more important for researchers to communicate to the regime that their work contributed to political aims, even if they were unable to guarantee any immediate success.

The argument that research had its own temporal logic was also present in research fields devoid of a science-technology nexus. In 1943, Joachim H. Schultze, professor of geography in Jena, expressed the belief that science ought to be one step ahead of the demands of the day. He defined fundamental research as “general research regardless of its practical application and regardless of the benefits of everyday life” (J. H. Schultze 1943: 197). He described research in geography as the task of depicting the overall research areas in the discipline, which included topics as diverse as the earth’s surface, landscapes, and the cultural and demographic depiction of countries. The central aim of Schultze’s article was not, however, to protect a self-referential concept of science, but rather to praise the utility of geography in general as well as the research carried out thus far for the purpose of warfare. Referring to historical examples of the huge political and economic interest in geography, Schultze argued that science, rather than being left to its own devices, needed both a societal mission and interest from the public. He advocated the idea of a central German geographical institute which would carry out fundamental and goal-oriented research for the state and for economic purposes. Schultze called for the combination of fundamental and goal-oriented research for an epistemic reason: research needs time and the future utility of scientific outcomes is not foreseeable as readily as future societal needs (J. H. Schultze 1943: 201). Thus, the term fundamental research stood for sustainable knowledge with potential benefit, or a sort of stock of knowledge (Ziegelmayer 1936: 253; Stock 1938: 150–151; Brüche 1944: 113).

### The Discursive Strategy of Fundamental Research and the Reassessment of German Science in the Nazi Period

Over the last decade, German science and its research endeavours under National Socialism have undergone a historical re-evaluation, namely within two major projects on the history of the Kaiser Wilhelm Society and the Deutsche Forschungsgemeinschaft (DFG, German Research Foundation). The focus has shifted to some extent from the effects of Nazi ideology and the participation of the humanities, anthropology, and medicine in racist and eugenic policies, to the hard and technical sciences that contributed to the military and economic goals of the Nazi regime. Whereas former studies stressed the negative effects of Nazi science policy, such as, the international isolation of the German scientific community, the experience of being cut off from raw materials required by the experimental sciences and the focus on substitute research as a result of a policy of autarky, and, since 1933, the incredible loss of excellent researchers as a result of racist science policy, recent studies present a more differentiated picture of science under the Nazi regime when focusing on research output and technical innovation.

Despite the regulatory claims of the Reich Research Council and the German Research Foundation’s loss of autonomy, recent studies show that researchers were still able to shape research policy according to their own interests. Provided that researchers showed a political affinity to the Nazi regime, scientific reputation and peer review continued to define the allocation of research funding (Flachowsky 2010). In particular after 1942, the year in which the Reich Research Council was reorganized and military technical equipment assumed greater importance in the German war effort, it appears that the regulatory claims of German research policy finally gave way to a more efficiency-oriented policy. As Mitchell Ash puts it, normal science existed throughout the Nazi period (Ash 2006: 34–35).

In this reassessment of German science and scholarship, the question of whether Nazi science policy led to a shift in focus from *Grundlagenforschung* (fundamental research) to *angewandte Forschung* (applied research) plays a crucial role. Recent studies provide evidence that fundamental research and applied research did not work as clearly demarcated, transdisciplinary, and supertemporal categories. Current studies on the history of the natural sciences during the Nazi period attest to a continuity of – what they call – fundamental research. Some studies suggest that German professors adjusted to the new conditions by combining applied research that accorded to political and economic requirements with fundamental research that earned greater appreciation within the scientific community in their projects. Although full professors apparently still honoured the ideal of pure science, most of them had contact with industry as individual consultants and/or via collaboration. Other studies identify fundamental research in especially applied contexts such as armament and defence research, but also in economically promising research fields such as metals research and polymer chemistry (Luxbacher 2010; Erker 2010; Flachowsky 2010; Epple 2002: 318–322). In the case of metals research, Günter Luxbach differentiates between research on the composition of metal, which was labelled as fundamental research, and research that tested the technical properties of metals, which was known as applied research. In contrast to this classical distinction between the quest for knowledge of nature and the quest for technological progress, Paul Erker describes polymer chemistry as a discipline that strove to combine these two motives. Erker employs the label of basic research for a heterogeneous and innovative research policy. Thus the meaning of fundamental research differs in historical studies on the natural sciences, not least because these studies investigate different disciplines.

By countering older historical interpretations that see German science in decline since its political instrumentalization in 1933, the main thrust of these recent contributions is, of course, that the Nazi’s war and policy of autonomy did not cause the profile of academic research to change overall. The insight that fundamental research went hand-in-hand with goal-oriented research is a novelty only if we analyse science on the premise that basic and applied research constitute two fundamentally different forms of research. Most of the historians quoted above still do not question the distinction between basic and applied research. The long-established categories still appear to be so self-evident that these authors do not feel obliged to define them explicitly for the specific research fields upon which they focus. Moreover, most of them still fail to reflect on how scientists employed terms such as *Grundlagenforschung* and *Zweckforschung* during the Nazi period.

Only a few of these historians have reconsidered their analytic vocabulary in light of new evaluations of the Nazi period. Moritz Epple, for instance, no longer believes in the opposition of the terms basic and applied. As in recent propositions in the philosophy of science, he suggests that we should speak of application-oriented fundamental research within the context of Nazi science (Epple 2010: 213). Another interpretation suggests that as German professors were increasingly involved in applied research, the use of the term fundamental research was merely symbolic, for the purpose of scientific reputation (Wagner 2010: 26–27, 33). Surprisingly, semantic sensibility is on the rise when it comes to discussing the aftermath of the Second World War. Within this context, the use of the term fundamental research is more often identified as a *simply rhetorical* strategy deployed by German scientists in order to retrospectively downplay their involvement in the Nazi system. Carola Sachse argues that this strategy of moral relief also worked in the American context: it was supposed to dispel fear of German post-war science (Sachse 2010: 480).

So far, this analysis of the first half of the 20th century has shown that the new terms fundamental and basic research initially emerged in mission-oriented or technical research fields. In Germany the concept only gained importance since the 1930s when research had to meet high political expectations. With regard to the historical context of the Nazi regime, the results suggest that the interpretation of a simply rhetorical strategy, whether as a strategy of individual moral relief or as a professional strategy for protecting a scientist’s guaranteed freedoms, is not entirely convincing. Because many German scientists demonstrated their commitment to the Nazi regime by offering their research services, the terms fundamental research did not serve to protect the old intrinsic ideal of science. In a period when the political expectations placed on science were high, the terms expounded instead the experience that scientific progress and procedures leading to exploitable results were difficult to predict.

## From Knowledge Sustainability to Purity Discourse: US Science Policy Between the Second World War and the Cold War Period

As the rise of basic research as a pivotal keyword in science policy during the post-war era was not peculiar to Germany, it is now time for a more comparative perspective. Although the two terms fundamental and basic research had gained greater currency throughout the 1930s in US science and, more generally, in science throughout the English-speaking world than had the term *Grundlagenforschung* in German science, they had not yet spilled over into all the different disciplines.^12^ Analysis of the journal *Science* demonstrates that, at that time, the use of these terms was still limited to biology (agriculture as well as studies on vitamins and proteins, which attracted pharmaceutical companies, also employed these terms), industrial research, and engineering. Once again, the terms denoted long-term studies focusing on fundamental problems in biology, chemistry, or physics emerging within the context of technical and application-related questions. The term fundamental research did not constitute an antonym to applied research; it was not part of a boundary discourse. In 1942, the research administrator of the US Department of Agriculture described basic research as follows:

In all these cases, either basic research precedes the practical applications of science, or a certain amount of this kind of research is found to be necessary somewhere along the line to clear-up obscurities that block further progress. … the point I am making is that in research there is no single road to practical results. If we keep our eyes constantly and exclusively on what seem to be immediate needs, we miss some of the richest fruits of scientific work – the fruits that grow from the discovery of important fundamental facts. … The emphasis I have given to basic research and freedom of inquiry does not mean that we should pay any less attention than we do to homely experimentation directed toward solving everyday problems. (Auchter 1942: 287, 288)

In the case of engineering, the concept of fundamental research largely represented the ongoing process of the scientification of technology (Gibb 1937: 233–234; Jewett 1944). Institutes such as the Mellon Institute of Industrial Research at the University of Philadelphia, which defined itself as a link between science and technology (or in the words of Edward Weidlein “between the world of science and the industry”), used the term fundamental research as a general label for their projects and training (Weidlein 1935: 562).

In light of these examples, the argument that the new concepts reflected the increasing expectation that science should be beneficial to the economy and to society as a whole also applies to the US case. Scientists were aware of the epistemic and institutional challenges to research that the 20th century brought forth. As a result of the increasing commercial potential of fundamental research, patents became a major issue at US universities quite early on. In contrast to the German universities, where the right to hold patents appeared to be considered part of the individual academic freedom of German professors (at least until the rise of the Nazi reign),^13^ the American land-grant colleges introduced patent regulations as early as the 1920s. Nevertheless, the administrative, legal, and ethical problems of patenting within institutions of higher education remained a controversial issue over the next few years (Potter 1940).

After dealing bit-by-bit with the shifting situation of science in the early 20th century, the Second World War marked an incisive and formative experience for the scientific community. When the US entered the war, the national mobilisation of science acquired the same level of importance there as in the other warring countries. In the early 1940s, the US debate on the effects of wartime revolved first and foremost around financial redistribution in science. The US universities, which depended mostly on private donations, were considered to be the losers in this process. In 1941, the long-standing idea of a federal fund that aimed to guarantee research funding on a regular and permanent basis was reignited. Although the financial crisis of the American universities had begun earlier during the Great Depression and bore several failed attempts to secure federal support for academic research (K. T. Compton 1934; Geiger 1986: 246–255), proponents of this initiative blamed the war for worsening the financial situation of the universities and diagnosed a crisis in fundamental research (Blakeslee 1941).

Those advocating federal support argued that a new form of funding was necessary because research in the basic sciences, that is in basic disciplines such as physics or chemistry, laid the indispensable foundation for future benefits: “We are all familiar with the material conveniences and comforts which science has given us, but we often forget the original patient, fundamental research which made them possible and will be the basis for future advances” (Robbins 1941: 8). As the concept of fundamental research had thus far denoted only research with a clear reference to application, the novelty here was the fact that the supporters of such a fund classified the entire endeavour of academic research at universities as fundamental research. Moreover, the lack of financial support for the universities was in opposition to the better funding of industrial and governmental research, which was only supposed to favour research that could demonstrate the prospect of immediate benefits (Robbins 1941).

### From Wartime to Peacetime: Vannevar Bush’s Plans for Transforming Science Policy

During the war, scientists had discussed the future conditions of science (Science 1942). At the end of the war, plans for a new science policy were already on the table. In the literature on research and development policy, funding for basic research and the dissociative model of basic and applied research in the post-war era are still inseparably linked to the name Vannevar Bush (Braun-Thürmann et al. 2010: 17). The MIT professor for electrical engineering served as presidential science adviser and, in particular, as chairman of the National Defense Research Committee and director of its successor organization, the Office of Scientific Research and Development. While coordinating the American military research programmes, including the Manhattan Project (the project devoted to constructing the atomic bomb), he began to make plans for a federal peacetime science policy. Based on the negotiations of four scientific committees (a Medical Advisory Committee, a Committee on Science and the Public Welfare, a Committee on Discovery and Development of Scientific Talent, and a Committee on Publication of scientific Information), in July 1945, Bush presented guidelines for future governmental promotion of scientific activity in the natural sciences and in medicine to the public. As well as providing financial support for academic research and junior scientists in the natural sciences, the proposals encompassed a reform of patent law and tax incentives for industrial research, the promotion of medical research, the plea for open science by fostering international exchange and strategies of declassification, and, finally, the sponsorship of basic research on military matters. Bush’s report “Science—The Endless Frontier” essentially sought to institutionalize federal science policy on a permanent basis (Bush 1945).

We should interpret his draft against the background of the war experience. The Second World War had demonstrated, once again, the importance of research for society and the fast-growing need for scientific knowledge. During the war, scientists and engineers had found that the search for technical innovation in the service of national defence spawned new questions and new problems for the natural sciences, the implication of which was long-term research. Given the immense expectations concerning immediate results within the context of warfare, some scientists feared that researchers would no longer be able to meet the demand of new knowledge for technical development (Simons 1943: 391). Despite the achievements made during the war, researchers warned of an exhaustion and future shortage of scientific knowledge: only by exploiting existing knowledge, they claimed, had it been possible to invent penicillin and radar, two of research’s major wartime success stories. In other words, there was a fear that the equilibrium between the production of scientific knowledge and its application would be disturbed (Bush 1945: 5, 8). The argument of knowledge sustainability thus became also important within the US community of scientists facing the war-time conditions of research.

This scarcity anxiety also applied to personnel resources in science (Barton and Burnham 1943: 176; H. S. Taylor 1944: 250). Bush’s report criticized the fact that, due to radical recruitment practices, the shortage of scientific personnel in the US was greater than in other countries (Bush 1945: 19). Bush’s colleagues, such as the Nobel Prize winner Arthur H. Compton, believed that the training situation and the support afforded to fundamental research^14^ at the universities were even worse in the US than in Germany (A. H. Compton 1945: 208). A lack of scientifically trained researchers also posed a problem for science-based industry. Thus the four committees suggested programmes for fostering scientific talent that included the generation in uniform returning from the war, particularly through doctoral fellowships for basic research.

In spite of this crisis and the discourse of epistemic and personnel shortage, wartime research efforts had, after all, strengthened the position of science in society. As the US government had spent more money on science throughout the course of the Second World War than ever before (Bush 1945: 82), scientists had a particular interest in perpetuating this federal commitment to science in peacetime. Since the US were traditionally characterized by less state intervention and a scientific infrastructure based largely on philanthropy and private donations, the federal support for academic research and training had been much lower than in Germany or in other European countries. It thus became necessary to legitimize the regular government funding envisioned through science’s role in the overall welfare of the nation. The Bush report justified the government’s obligation to support basic research in three ways. Firstly, medical research would improve public health. Secondly, research would advance the overall public welfare, which was almost synonymous with economic growth and job security due to innovations and new products. And finally, long-term civilian research promised to give the US a technological edge in armaments which was supposed to guarantee national security. Only then did basic research become a real keyword in research funding. And the metaphor of “basic” did the trick; by laying the basics for all kinds of future benefits, the federal government financed basic research as for the common good.

Bush’s proposal also reacted to the organizational conditions of wartime research, in particular with regard to security restrictions. The problem of secrecy policy had already been discussed openly during the war (K. T. Compton 1942: 28). Bush’s report called for the prompt release of classified research after the war. This request also involved a secrecy strategy in which projects were split up into small, isolated research groups, each of which worked on a specialised problem without the opportunity for any kind of exchange between them.^15^ It is worth mentioning that the work on these specialized problems within isolated research groups was sometimes denoted as fundamental or basic research during the war (Simons 1943: 392), which indicates that Bush’s use of the term deviated from the former understanding. In order to near the ideal of open science once again, Bush believed that the federal government was also obliged to encourage publication, international communication, and cooperation following the war. In general, the report restricted the role of federal science policy to financial support and the provision of coordinating infrastructure (Bush 1945: 22–24). Bush sought to prevent the government and the military from continuing to pursue the managerial approach to science policy they had applied in wartime. Bush criticized the military leadership for being too narrow-minded, a characteristic that did not fit with his understanding of the speculative and multidimensional nature of research (Reingold 1987: 338–341). Failed attempts to establish federal research funding had already demonstrated that the majority of the scientific community disapproved of any governmental intervention in science (Geiger 1986: 255).

Other scientists shared with Bush the rising concerns over free scientific exchange toward the end of the war (H. S. Taylor 1944: 255; Jewett 1944: 3), but Bush’s report was the first to link the relatively new notion of basic research with an institutional guarantee of scientific autonomy in such a close fashion. This also included his contrasting juxtaposition of basic research with applied research and development. By then, applied research had not been an antonym to basic research. Basic research thus not only meant that science should be freed from the burden of high expectations tied to immediately exploitable results; it also entailed the freedom of both inquiry and scientific communication. The different agendas and arguments – the strengthening of US universities as research and training institutions, the switch from war to peace, and attracting federal support for science in the name of national welfare – converged in the report’s recommendation to enhance the universities and non-profit research institutions as centres of basic research.

Following the release of the report, US scientists, politicians, and industry representatives entered into controversial discussions on various aspects of Bush’s proposals, which delayed the establishment of the National Science Foundation for nearly five years. The patent issue, the suggestion that the social sciences be excluded from federal support, the uneven distribution of excellent research universities in the individual states coupled with the corresponding problem of how to fairly allocate federal funding, and, finally, the envisioned scientific expertocracy within the federal foundation proved to be particularly delicate subjects. Bush’s proposal faced opposition, in particular from the military, liberal and democrat activists, and even from scientific colleagues. At the universities, which stood to benefit most from the funding, some scientists considered the plea for basic research as restricting their funding and research habits, which included contract research for industry or the army. There is no need to go into the details of this debate here since a mass of literature has already revealed these conflicting institutional interests and the political dimension of the controversy on science policy in the early years following the Second World War (Kevles 1977; Reingold 1987; Owens 1994; Zachary 1997: 218–239, 249–260; Guston 2000; Dennis 2004). Most interpretations allude to Bush’s political conservatism aiming at the restoration of the pre-war political order. More generally speaking, studies on post-war research policy have so far presented a mainly political reading of these debates, which essentially revolved around the issue of more or of less intervention of the federal government into research. Even Roger Pielke’s (2012) current interpretation confines the concept basic research to this political dimension: in his view, the symbolic capacity of the term accommodated the conflicting parties, striving for the organization of science by the federal government on the one side and the autonomous organization of research by scientists on the other side, by promising potential utility.

What this analysis can add to the previous literature is a more nuanced interpretation of the conflicts within the scientific community. I argue that the scientific controversy over Bush’s “Endless frontier” partly stemmed from differences or even misunderstandings in the semantics of basic research. Although Bush developed the concept from the common discourse on knowledge sustainability, he added new semantic dimensions that had to meet multiple requirements of a new funding programme. In what follows, I demonstrate how the various problems in research organization and the overall political climate during the Cold-War period were turning this sustainability discourse by and by into a purity discourse.

### Bush’s Definition of Basic Research: The Beginning of a History of Misconceptions?

Bush’s report marked a semantic shift in basic research that made a clear break with existing practices and notions in order to reorganize research in the post-war period. His specific use of the concept of basic research thus gave rise to misunderstandings and confusion. Moreover, the history of basic research in the second half of the 20th century has been characterized, in part, by these misunderstandings, which in the long run evoked anachronisms over which science and technology studies are still puzzling today. Bush’s short definition of basic research as “research performed without thought of practical ends” (Bush 1945: 13), still singled out by most studies (Stokes 1997: 116; Godin 2005b: 265; Popp Berman 2012: 21), definitely contradicted the original understanding of basic/fundamental research in the context of application. Even more so, this reduction fails to represent the conceptual range of basic research in the report and the wider debates that took place right after the war.

Bush’s definitions of research, science, and applied science confused his peers and even one of his closest companions James Conant, who reflected critically on the new conceptual boundaries (Conant 1948). Representatives of national or military laboratories felt particularly compelled to argue against an institutional separation of basic and applied research. A member of the Naval Ordnance Laboratory, for instance, argued that “the naval laboratory programmes make it necessary for us to carry on basic research in certain parts of certain fields simply because no other agency is interested in, or has the facilities for, doing this work” (Bennett 1946). Bush’s peers in engineering were certainly confused by the different ways of denoting basic research. Universities specializing in the applied sciences and engineering, such as the California Institute of Technology (Caltech), understood basic research – inseparable from the overall pragmatic goal of inventing new technology – as an integral part of modern engineering (DuBridge 1959: 109–110).

Following the publication of “Endless frontier”, scientists and other policymakers tried to differentiate and redefine the concept of basic research, which indicates that Bush’s understanding of basic research was not taken for granted and that researchers struggled with it because it did not fit the existing research landscape. John Steelman, science adviser to President Truman, for instance, divided basic research into two subcategories: firstly, fundamental research defined as “theoretical analysis … directed to the extension of knowledge of the general principles governing natural or social phenomena”, and secondly, “background research” defined as “systematic observation, collection, organization, and presentation of facts using known principles to reach objectives that are clearly defined before the research is undertaken to provide a foundation for subsequent research” (Steelman 1947a: 6). Others tried to introduce a distinction between “fundamental research, which leads to an understanding of the laws of nature, the discovery of new facts and laws, and the theoretical development”, and “basic research as it applies to industrial or military development involving basic studies of the fruits of fundamental work to determine their potentialities antecedent to application” (Leob 1946: 540). An industrial researcher defined basic research as an intermediate category, which he located between pure research as “inquiry after knowledge for its own sake” and applied research as “the investigation carried out in response to immediate, direct, and obvious needs” (Spaght 1955: 785). The gradual emergence of new variations such as “mission-oriented basic research” indicates, at least, that the criterion of intention, whether utility-oriented or not, became problematic in the long run (Tuve 1959: 174; Kistiakowsky 1966: 18).

While all these redefinitions can be interpreted as a claim to reintegrate application goals into the concept of basic research, Bush himself actually did not exclude the idea of mission-oriented research. Nathan Reingold sees “the pursuit of new knowledge” – and not the pursuit of knowledge for its own sake – as the real core of Bush’s notion of basic research. Reingold further refines his interpretation by quoting Bush’s argument that “there is no specification as to whether the knowledge is or is not of direct utility” (Reingold 1987: 305). That sheds a very different light on the story of basic research. The importance of new scientific knowledge becomes even clearer if we take into account Bush’s metaphor of “the endless frontier”, which emphasized the cutting-edge role of scientific research. It thus placed scientific problems at the border of the unknown, reflecting the uncertainty of scientific outcomes and their long lead time in a world increasingly reliant upon scientific progress. Not least, Bush’s outline for a federal funding programme entailed financial support for basic research (long-range scientific research) on military matters.

Nevertheless, some of Bush’s contemporaries interpreted the report as an attempt to return to the old intrinsic ideal of pure science. They criticized the report for favouring a selfish notion of scientific autonomy that did not respond to any societal or economic needs (Shepard 1946).^16^ My analysis thus far reveals that the discursive strategy of basic research initially aimed to acquire regular financial support from the government by promising utility in spite of the uncertainty of scientific research. This happened to conform to the interests of the scientific profession, so long as this support did not affect the ideal of open science. In the long run, however, the allegation of a return to an ancient pure-science ideal proved to be true. As the promotion of basic research continued, the sustainability discourse was transformed into a purity discourse, which revealed aspects of a past notion of science deemed to have been superseded in the 20th century.

After having become the spearhead of scientific endeavour, surprisingly enough American researchers looked back towards continental Europe. Post-war proposals for higher education in the US idealized the European university system and its humanistic tradition by associating it with democracy (Bender 1997: 4–5). The old model of the European research university and its success story in basic disciplines such as physics and chemistry became a role model. According to Bush’s report, the arguments for reinvigorating the university within an increasingly pluralistic research landscape were twofold: immense need for scientifically trained researchers and the demand for scientific autonomy.

American scientists had repeatedly commented on the short-sighted focus of armaments research and on the threat Hitler’s regime implied for the freedom of science, presenting the liberal democratic order as the only safeguard for the fundamental principles of science (Fosdick 1934: 380; Simons 1943: 392; Goudsmit 1947: XI). The debates on lessons to be drawn from the war experience, however, demonstrate that American scientists were chiefly worried about the threat to scientific freedom with regard to their own national conditions (H. S. Taylor 1944: 255; Goudsmit 1947: 232–246). Bush’s report blamed previous federal policy – from the Morill Land-Grant Colleges Act to the more recent practice of contract research – for being primarily interested in immediate benefits. The report argued that, due to an alleged inclination towards more utility-based research, the American nation depended entirely on the European production of new scientific knowledge (Bush 1945: 2; see also Astin 1959: 146–147).

By reproaching the societal and governmental focus on the utility of science, Bush’s sustainability argument was just about to tilt over towards the purity ideal of science. This explains why some scientists initially reclaimed the application aspect of research. The related aspect of academic autonomy, however, met with the approval of most scientists. Aside from the politicians supporting a science policy agency like the Democrat Harley M. Kilgore (1945: 636), only a few scientists argued frankly against the anxiety about governmental interference by pointing out that the increasing social and economic demand for scientific research and the necessary political coordination of research in response to these needs were facts which scientists in the 20th century had to accept (Dunn 1945). Although the final establishment of the National Science Foundation turned out to be a compromise for all parties having negotiated this new form of federal science funding, Bush’s altered definition of basic research, in the end, became accepted.

At the end of the 1950s, after the National Science Foundation had been operating for several years, scientists continued to criticize the low federal base rate for basic research in comparison to that of contract research in the Department of Defense, which was twice as high (Elvehjem 1959: 94; Waterman 1959: 26–27). Some deployed the sophisticated argument that many projects were not truly basic research, but actually mission-directed basic research. In fact, the Korean War had meanwhile intensified the Cold War conflict and the Soviet’s launch of the Sputnik satellite turned the ideological competition between West and East into a science and technology race (Tuve 1959: 173–176). As a consequence, basic research stood primarily for federally financed *academic* research – with or without any concept of practical use.

From the outset, the various drafts of the documents formally establishing the National Science Foundation included fellowships for graduates and junior scientists, so that the concept of basic research was closely linked to training scientific talent (Steelman 1947b: 29–30). From the late 19th century onwards, the modern research university inevitably moved further and further away from the traditional concept of a university as a specialized institution of higher education that excluded any notion of material benefit or practical aims. Yet the post-war debate on support for basic research led to a new version of the old boundary discourse of pure versus applied and theory versus practice. At a major symposium on basic research in May 1959, one representative of a private technical university reasoned that the

most difficult questions arise as to what is fundamental research, what is practical development, and which projects could be more appropriately done in commercial laboratories. … One useful criterion which helps many decisions in this field is that to be acceptable in any area a research program must be one which is consistent with and contributes to the educational program. This means it must be one in which graduate students can participate. This means, among other things, it must not be ‘classified’, either for reasons of trade secrecy or military security. (DuBridge 1959: 109–110)

In the discourse among academic teachers, the ideal of training “good scientists” was not compatible with military or other contract research (Elvehjem 1959: 94). Even engineering sciences felt compelled to adopt pure-science ideals whereby profit and research projects with self-serving interests should be taboo in institutions of higher education as long as they were part of scientific training. Given the fact that the growing number of military-related research projects at universities during the Cold-War years often included doctoral students, these statements certainly did not mirror the actual practice in the higher education of engineers (Dennis 1994). They rather seem to reflect the increasing uneasiness with the security guidelines related to contract research for the armed forces and the increasing number of military-related research projects.

The call for new knowledge through basic research in the post-war era also reached industry. Big companies such as DuPont or the Bell Telephone Company, which could afford their own well-equipped laboratories, intended to expand their participation in basic research after the war had ended (Fisk 1959). However, since economic rationales entailed selecting projects that were most likely to lead to innovation, these companies welcomed the idea of the federal government funding riskier projects to be carried out at the universities (Greenewalt 1959: 130). After all, failures and deadlocks – all more or less inevitable parts of the scientific production of knowledge – would cause costs they wanted to avoid. Furthermore, industry representatives appreciated federal support for training the future generation of researchers they needed. This division of labour was financially promising for companies as “a technological savings account” (Greenewalt 1959).

While the amount of research carried out in direct response to economic and military demands had increased tremendously since the Korean War (Killian 1959a: 122), the university was meant to become a sort of reservation for long-term basic research within a changing research landscape. Academic freedom in the second half of the 20th century largely sought to liberate science from over-the-top societal expectations. Protecting scientific research “from the insistent demands of applied research” became a central argument deployed by scientists as well as industry and politics (Weaver 1959: XIV; see also Greenewalt 1959: 128). Yet what was initially intended to protect scarce knowledge resources could, in the long run, transform into an ideal of purity. The university was granted the status of a reservation in the midst of a rapidly changing research landscape in order to protect science against excessive expectations and thus guarantee the open development of scientific knowledge. With the status of reservation, however, also came the danger that research conditions be artificially conserved, making it difficult to respond to changes in scientific practices.

### The Revival of 19th-Century Epistemic Norms and Virtues

The shift from a discourse of knowledge sustainability to a discourse of purity affected the epistemic concepts of science in particular and, in so doing, appeared to hark back to ideas coursing in the 18th and 19th centuries. First and foremost, this shift concerned the relationship between the natural sciences and technology. Although Vannevar Bush himself dealt with basic questions in mathematics – a central basic discipline in engineering – as well as with construction design in his own research, his proposals ended up reviving the old distinction between nature and technology because they made the distinction between engineering, on the one hand, and the natural sciences, on the other.

Historians have explained this distinctive position on the natural sciences with Bush’s personal concepts of administration and his ideas about achieving excellence in science through specialized researchers, based, of course, on the premise that the rationale of open science would guarantee the unhindered diffusion of knowledge for the benefit of technical progress (Reingold 1987: 306–307). This relapse into outdated concepts of science, however, cannot be reduced to the personal preferences of Vannevar Bush. It should instead be seen as a broader academic phenomenon, which began as a move to counter the increasing demand on science for immediate benefits that reached its height during the Second World War, before finally turning into a political programme in the West, nestled within the ideological competition of the Cold War.

In order to protect basic research in the natural sciences, academic experts wanted these disciplines to steer clear of any kind of technical development. As Alan T. Waterman (1959: 28) proclaimed in 1959, “the growing applications of physics, chemistry, and mathematics should be shifted to engineering departments and kept out of the regular science departments”. In other words, from the point of view of the natural sciences, applied research primarily meant research that sought to yield future technology.

An oceanographical study carried out within the context of naval research in the late 1940s and early 1950s reconfirms this one-dimensional understanding of applied research in contrast to basic research. The US Office of Naval Research was a staunch supporter of basic research in oceanography, yet the question of secrecy revealed that the Navy and scientists differed in their classification of basic and applied research and in their notion of utility. Oceanographers defined their investigations of the topographical features or meteorological conditions of the ocean as basic research as long as it did not expressly serve the development of technology destined for use by the Navy. The Navy, however, developed “a more sophisticated definition of basic research that would take its operational nature into account” and demonstrated strategic utility of geography for military purposes (Hamblin 2002: 27).

This purification of the natural sciences even affected the existing research vocabulary. Science policy experts tried to find new labels for research fields in engineering formerly classified as fundamental or basic research. The term “analytical engineering” is a good example of this renaming practice (Killian 1959a: 122). Moreover, in the debates revolving around basic research in the post-war era, the whole attitude towards technology appeared to become more ambivalent. In the 1950s, the National Science Foundation still justified the support for basic research primarily by the goal of enhancing technical progress. At the same time, it became ever more common for statements on science to conclude with a declaration bearing the motivating force behind scientific endeavour; the pursuit of knowledge for its own sake and the quest for truth became the appendix of federal science policy (Waterman 1959: 37–40; Astin 1959: 154).

Researchers in innovation studies have associated post-war research policy with the “linear model”, that is with a linear trajectory from basic research in the natural sciences to technology (Edgerton 2004). Implicit in the new policy of basic research was a renaissance of the older epistemic notion of an asymmetry of knowledge and, by association, the scientific preference for research led by theoretical questions. Particular support for basic research in the natural sciences was grounded in the hope that a few basic discoveries would be sufficient to significantly broaden the potential for technological application (Elvehjem 1959: 98). In the process of striving for the endless frontier of the unknown, the idea of major theories in the natural sciences came to be the ultimate driving force of scientific progress and thus a further argument for supporting basic research.

Even representatives of industrial research endorsed the orientation of academic research towards theory in order to provide mutual benefit:

[T]he existence of even a crude and preliminary physical theory and the heeding of it in the expectations and patterns of operation of scientific work would permit coupling of the individual, uncommitted, undirected researcher to the general objectives of economic and social programs. … In the still regrettably small list of findings from basic scientific research which have been quickly and directly connected with large advances in technology and useful operations are several important examples. In these, the really new idea came out because a unifying theory had displaced the true possibilities – the wide range of means rather than simply the ends themselves … (W. O. Baker 1959: 54).

This hierarchical and linear notion of knowledge production contrasted with a more dynamic understanding of the relationship between fundamentally theoretical questions and approaches that started out from a concrete problem of application. Although the professional self-image of academic superiority certainly continued to have an effect on epistemic ideas and norms in the late 19th and early 20th centuries, shifting research practices had already begun breaking up this static epistemic model. As the special support of basic research and its distinctive position within the different research activities was beyond dispute in the late 1950s, representatives of industrial research or national laboratories only casually mentioned the mutual reinforcement of theoretical and application problems they encountered (Astin 1959: 145, 151; Fisk 1959: 160–161).

Debates on basic research eventually revealed another old epistemic ideal referring to the intellectual qualities of researchers and to research conditions that encouraged scientific creativity. New (federal) support for basic research initially focused on individual researchers in order to foster “the development of the individual scientist” (Waterman 1959: 34; see also Weaver 1959: XI; Greenewalt 1959: 128–131; Morison 1959: 230). Experts esteemed individual creativity as the main property of outstanding scientists, enabling them to move forward into the unknown. The free flow of unconstrained intellectual creativity was thus defined as basic research. Not least, the financial relief stemming from regular federal funding was well received as a guarantee of intellectual freedom (Tuve 1959).

This particular position was backed up by the revival of old academic virtues. “[T]ruly ‘basic research’ was driven by a passionate love for knowledge. Basic research thus meant ‘support for ideas’ in the first place” (Tuve 1959: 174, 175; see also Waterman 1959). This definition of basic research tended to be averse to technology. Furthermore, the hierarchy of basic and applied research implied the moral superiority of academic research over benefit-oriented industrial research, even on the personal level of researchers (Elvehjem 1959: 94–96). In the end, the epistemic virtue of disinterestedness – according to Robert Merton one of four imperatives of modern science – got mixed up with social and moral values.

This deep appreciation of individuality was partly a reaction to the growing experience of scientific teamwork, which had become common within large military or industrial research projects. Individual creativity contrasted with the conservative atmosphere of research groups, which tended to object to fresh, radical ideas (Waterman 1959: 30; Tuve 1959: 176). Even those involved in industrial research highlighted the advantage of academic research because companies were only able to offer limited space for the individuality of their researchers. Furthermore, the freedom of investigation was supposed to be a special incentive for academic research – an incentive that had to compete with the high salaries and the technologically well-equipped laboratories in industrial research (Elvehjem 1959: 96–97). Praise for individuality in science, however, derived partly from the ideological value of individualism in Western civilization. The first director of the National Science Foundation, Alan T. Waterman, put it like this: “Surely one of the great assets of democracy is the encouragement of individual initiative” (Waterman 1959: 25).

### Democracy at Risk: The Ideological Role of Basic Research in the Cold-War US

The ideological potential of the basic-research concept contributed significantly to the shift from a discourse of sustainability to one of purity. Politicians, for example US President Dwight D. Eisenhower, translated the new science policy directly into political slogans such as “Science: Handmaiden of Freedom” (Eisenhower 1959). Politicians still placed great hopes and expectations on science as the pacemaker of technical progress, capable of securing national security, national welfare, and prosperity. At the same time, their support of basic research enabled politicians to praise academic freedom as an overall value of liberal Western society. In addition to this, federal funding for basic research, defined as support for individual initiative and creativity, symbolized the individualism within democracy (Waterman 1959: 25). As a collective symbol bridging the gap between scientific and public discourse by the polysemy of metaphors, basic research offered a true ideological surplus. Politicians further contrasted the “limited or local application” within mission-directed research with the universality of basic research designed to “benefit all mankind” (Eisenhower 1959: 137). Leading the technological race with the launch of its Sputnik satellite, the Soviet Union then stood for an application-oriented understanding of science in the service of communist goals, whereas the Western argument pertaining to the universality and openness of basic research claimed ethical superiority.

During the 1950s, this high praise for free basic research stood in opposition to the high percentage of projects funded by the military and the increased demands for secrecy imposed on large areas of research in physics or other fields relevant to military projects by US security policy. It is telling that, in 1951, Alan Waterman, first director of the National Science Foundation and former technical director of the Office of Naval Research, emphasized the role of science in the situation of national emergency in the wake of conflict with the communist world; in spite of the National Science Foundation’s basic research programme, he underlined the need for science to focus on urgent application problems (Waterman 1951). According to the literature (Forman 1987; Westwick 2000), patriotic mobilization among scientists was still high. Many classified their research voluntarily, or adjusted to political pressure for security by compartmentalizing research and forming classified communities. Although these strategies were supposed to guarantee as much scientific exchange as possible, secrecy meant that research largely took place within a national context.

Moreover, the debates in *Science* during the 1950s demonstrate that the secrecy policy and the effects of a dominating military grip on science gave more and more cause for concern within the scientific community. Scientists criticized the idea that the military had a “sophisticated understanding of the needs of basic research”, arguing, moreover, that “those branches of pure science that lack military appeal are as badly off financially as they ever were” (Phillips 1952: 440). In the early 1960s, military or military-related institutes, such as the Office of Naval Research, were still financing most academic research, in particular at prestigious universities (Leslie 1993). Against this backdrop, the political reading of basic research was not merely an aspect of portraying the US as a liberal society to the outside world. The debate on basic research also reflected, more controversially, the internal effects of the cold war on research. The debate was embedded in a more general intellectual discourse on the consequences of the predominant security policy and the growing power of the military for democratic society (see, for example, Shils 1956: 176–191).

Eisenhower’s statements demonstrated this growing ambiguity. In his well-known “Farewell Address” from 1961, the departing president, former supreme allied commander and president of Columbia University, warned against the growing power of a “military-industrial complex”:

[W]e must guard against the acquisition of unwarranted influence, whether sought or unsought, by the military-industrial complex. The potential for the disastrous rise of misplaced power exists and will persist. We must never let the weight of this combination endanger our liberties or democratic processes. We should take nothing for granted. Only an alert and knowledgeable citizenry can compel the proper meshing of the huge industrial and military machinery of defense with our peaceful methods and goals, so that security and liberty may prosper together. (Eisenhower 2003: 414)

Eisenhower construed financially attractive contract research as a threat to the academic “fountainhead of free ideas”. More importantly, he warned against the menace to public policy and civil society of a new “scientific-technological elite” (Eisenhower 2003: 414–415). Although Robert Merton had already stressed the similarity or affinity between open science and Western democracy, in the late 1950s and early 1960s Eisenhower and other politicians identified science as a threat to democracy when a close connection between science, the military, and the economy remained intact (Wang 1999b).

Along with the attribute of universality, another of Merton’s four imperatives of modern science, the notion of truth also gained importance in this ideological discourse (Waterman 1959: 39). The ideal of truth had already been part of the ideological fight against fascism during the Second World War when researchers emphasized that science offered more than technical applications: “American science therefore has an especial duty to keep aflame the torch of free research for truth, which is dimmed or gone out in so many lands” (Blakeslee 1940: 592).

As the natural sciences had needed a long time to set themselves apart from an understanding of science dominated by natural philosophy, the revitalization of the idea of universal truth appears anachronistic. In the 19th century, the natural sciences developed a mechanical and structural understanding of objectivity based on methodological processes that sometimes even stood in contradiction to the quest for truth and certitude (Daston 2000: 32–34). At the beginning of the 20th century, the quest for truth had something old-fashioned about it in a scientific era in which research was constantly doing away with established certainties.

### Coping with Ethical Dilemmas in the Cold-War Era

During the Cold War, however, the attributes of truth and universality were revitalized and became part of an effort to present science as a politically and ideologically independent authority in society. From the viewpoint of politics, science was able to act as a neutral authority upon which decision-makers could rely (Price 1962: 1105). Scientists themselves praised the idea “that science has something more valuable than its material gifts to offer. … Science can have no dogma, no arbitrary authority, no ‘party line’” (Sinnott 1950: 125). Scientific virtues of “objectivity, tolerance, reluctance to distort or suppress evidence, and willingness to accept sound logic and demonstrable fact” were transformed into political virtues (W. P. Taylor 1953: 449). At the same time, however, the position of impartial experts tended to be morally overloaded when scientists were meant to become missionaries of “reason and good will” in the fight against “falsehood and hate” (Sinnott 1950: 126; see also Szent-Györgyi 1957; Rapoport 1957; Weaver 1961: 259). In fact, the democratic framing of basic research and the revival of knowledge ideals in the tradition of Humanism led to a politicization of science and, as a result, basic research itself became part of ideology, namely Western ideology.

Historians have already pointed to the various ideological dimensions of science in the post-war period (Wang 1999a; Ash 2006: 30; for the social sciences and humanities, see Bender 1997). Some scholars from science and technology studies blame Robert Merton’s comparison of science in democracy with science in fascist and communist regimes for the misconception of scientific ideals such as autonomy and universality, a misconception that they have been trying to correct ever since (see the overview in Daston 2000: 18–20). But the societal, political, and ethical implications of the basic-research concept were embraced by the scientific community, even without sociological mediation.

After the atomic bomb was dropped on Hiroshima, the role of science in society certainly became more contradictory (Conant 1961: 6–13). While researchers had wholeheartedly praised the salutary benefits of science before Hiroshima (A. H. Compton 1940: 56), contemporaries noted afterwards that the “atom bomb once and for all explodes the ‘neutrality’ of technology” (Shepard 1946: 66). The promise of progress was only one side of the coin. Scientists became increasingly aware of the burden of responsibility in their own research. Some of them hoped to avoid this problem by pursuing more theoretical research topics. Others tried to take political action, such as the atomic physicists’ movement, which fought for civilian use of scientific knowledge and technological invention. But the anxious atmosphere during the Cold War period – anti-communist harassment and the increasing public fear of a new scientific-technological elite – aggravated the ethical dilemmas of post-war science.

A statement made by the physicist Julius Robert Oppenheimer, a leading figure in the Manhattan Project, about the debate on basic research indicates scientists’ uneasiness when they were faced with these dilemmas: “The argument that the quest for new knowledge, which is basic science, is ennobling, and the argument that the quest for new knowledge produces new knowledge which is useful to technology and thus to practice, are disturbingly separate and unrelated arguments. … Yet science and technology are symbiotic” (Oppenheimer 1959: 9; for a similar argument, see W. O. Baker 1959: 43–47). Oppenheimer seemed to suspect that the debate on basic research simply reflected these modern dilemmas. It is striking, but also telling, that he tried hard to avoid the dualistic semantics that characterized science policy at this time. Oppenheimer explicitly raised the political problems brought about by the powerful scientific culture of the 20th century. Taking the growing criticism toward scientists into account, the physicist believed that making the public understand research goals had become difficult. While the impact of science on society had increased tremendously, the fast growth of scientific knowledge and technical innovations made it hard for laypersons to judge issues in science policy. Oppenheimer feared that this asymmetry of knowledge between experts and the lay public weakened democratic political decision-making (Oppenheimer 1959: 12–13).^17^

The charges brought by the McCarthy Committee in 1954 against Oppenheimer relating to his opposition to the hydrogen bomb illustrate that scientists who were willing to assume responsibility for their research by taking political action had to learn the hard way that there was little room in the political climate of the Cold War to deal openly with these dilemmas of modern science (Bird and Sherwin 2005: 462–550). With regard to scientists of the progressive left advocating a more utility-oriented notion of science, Jessica Wang notes that “[a]lthough their views on the structure of postwar science were not directly responsible for their political difficulties in every case, these scientists and others who embraced a liberal-left politics of science were likely to hold other views that made them vulnerable to anti-communist attacks and excluded them from political influence” (Wang 1995: 166). In the mid 1950s, the National Science Foundation and the Academy of Science included the criterion of national loyalty into their peer-review system for unclassified research. Both organisations thus sought to avoid allegations of supporting researchers who were suspected of sympathising with communist ideas (Waterman 1960: 127; Committee on Loyalty in Relation to Government Support of Unclassified Research 1956).

The question of loyalty arose especially when it came to discussing technological application, as an official statement by the President of Associated Universities addressed to the Committee on Government Operations confirmed:

If a scientist expresses a strong view on some technological matter that may be contrary to the application of technology to current or to subsequent policy, he is open to the accusation of taking this view with the intent of deliberate subversion. … Moreover, secrecy prevents him from stating the essential technical grounds on which his view is based. Therefore, in the simple process of doing his job for his country well, he is open to damaging criticism against which he is permitted to produce little defense. (Berkner 1956: 784–785)

Given this pitfall, the discursive separation of science from technology provided a strategy to avoid the risk of being forced to go “politicking”, which gradually came to be considered as the “disease” of the project research dominating American universities at that time (Gates 1958: 234).

In this particular situation (the ethical dilemmas of the techno-scientific world, the fragile relationship between science and the public in democracy, and the ideological antagonism during the Cold War), the dissociation of the natural sciences from applied research and any practical application of scientific knowledge was thought to offer a strategy of individual, professional, and institutional relief: Firstly, a strategy that avoids assuming ethical responsibility for the changes caused by scientific knowledge. Secondly, a sort of self-protecting strategy that sought to avoid the direct line of political fire in a society entirely concerned with national security, the latter which produced an atmosphere of suspicion. And thirdly, a strategy of political neutrality and independence from any self-serving interests as a means of guaranteeing the institutional freedom of academic science and a self-regulating scientific community which, from a scientific point of view, was best capable of dealing with the open and often unpredictable process of epistemic progress. The scientific community retreated into a “satisfactory philosophy of ignorance”; as long as science was defined as institutionalized scepticism, it was still possible to maintain the belief in science or scientific knowledge as an indispensable value of modern civilization (Feynman 1955: 15).

### Conflicting Promises and Their Effects on the Public Image of Science

This neutral position secured the federal funding of research at universities in the US – something the universities had longed for since the 1920s. In return, academic researchers promised simply that science would lay the foundation for progress. They also offered their expertise to politics, thus acting as an independent authority over truth in a pluralistic, democratic society. The certainty academic scientists offered appeared to be especially welcome at a time in which society was driven by great anxiety. With regard to the outside image of the US during the Cold War, the universities’ role as reservations devoted to autonomous science served as a symbol for Western liberal society in the tradition of Humanism amid the great ideological competition, while simultaneously providing fig-leaf camouflage for the technology-based arms race. The post-war understanding of scientific autonomy was, in fact, the result of a broad process of the politicization of science arising from the growing importance of scientific knowledge for society.

Since there is, by definition, no clear solution for dilemmas, the strategy of basic research inevitably caused problems for the relationship between science and the public in the long run. Articles on this relationship and on topics such as the responsibility of science in the late 1950s show that public mediation between the needs of science and those of society became increasingly problematic (Killian 1959b: 136; Sayre 1961; Price 1962). According to Bender, this understanding of the autonomy of science, in particular the position of elitist experts and how they neglected their responsibilities, alienated science from society, evoked the impression of an academic ivory tower, and, finally, ended in federal budget cuts for academic research (Bender 1997: 8–12).

Moreover, I argue that the simple promises of truth and progress scientists had avowed to society covered the complexity and uncertainty of research dynamics as well as the tentativeness of contested scientific knowledge. Moreover, the authority of scientific objectivity and methodologically certified knowledge revealed its limitations during political negotiations on values and societal goals; the position of moral neutrality might bewilder the public. It could thus lead to disappointment, misunderstanding, and even to the loss of science’s integrity in the public sphere. Furthermore, the increasing interlocking of technology and the natural sciences was also hidden behind praise for basic research. Since technological innovation had become part of the natural sciences, questions of risk and utility had inevitably arisen and transformed themselves into political and ethical issues: Who will profit from the results? How do we manage risks?

Only few researchers at that time anticipated that the excessive expectations of and contradictory demands on research might turn the public against science (W. O. Baker 1959: 48; Dryden 1954). The shift from a discourse of knowledge sustainability to one of purity meant that the concept of basic research itself sent contradictory signals to the public: “The uneasiness of scientists on this score is revealed by the observation that, whereas they claim among themselves that their primary interest is in the conceptual, not in the applied, aspects of science, in public they justify basic research by asserting that it always leads to ‘useful’ results” (Dubos 1961: 1209; see also Daniels 1967).

In fact, the concept of basic research and the underlying linear model of innovation had already come under attack in the late 1960s and early 1970s. The long-term and highly speculative nature of scientific research was difficult to communicate to a public that expected economic prosperity and welfare here and now. Society’s disappointment backfired on the scientific community and stimulated a debate about the appropriateness of dissociating basic from applied research (Abelson 1966; Reagan 1967). Yet this crisis is another chapter in the conceptual history of basic research and goes beyond the scope of this paper.

Despite recurring crises, the concept of basic research functioned as a collective symbol for science policy over quite a long period of time. Moreover, the semantics of the new US science policy spread across the entire Western world. Ever since the National Science Foundation established a periodical survey of overall research in the US based on the categories basic research, applied research, and development (the final stage of innovation, when technologies or ideas are turned into marketable products), nearly all countries in the Organisation for Economic Co-operation and Development (OECD) adopted this classification (OECD 1976). Basic research and its corresponding categories were converted into enduring statistical realities that played a crucial role in budget planning within industry and in funding allocation undertaken by government bodies (Godin 2005b).

### Fundamental Research in the Federal Republic of Germany: A Brief Overview

In the Federal Republic of Germany (hereafter referred to as West Germany), fundamental research also became a key concept in science policy. The impact of the American role model on West Germany is quite obvious. Within the context of re-education and development programmes, those representing US science promoted their concept of science in democracy with its special focus on fundamental research in West Germany (Conant 1953; Bush 1954). However, the national characteristics of the German research landscape coupled with the historical burden of the Nazi past meant that the way fundamental research and its corresponding discourses were implemented differed to a certain degree from the American experience. I will briefly mention some of these Germany-specific characteristics in order to maintain a balance between the two national perspectives.

After the Second World War, the Allies assumed control of science in Germany with the intention of suppressing all further research activities relevant to the development of armaments. Allied Control Council Acts and the ensuing executive regulations specified by each of the Western occupation zones forbade any fundamental or applied scientific research with military relevance (Frowein 1949, 1950).^18^ It is remarkable that the crucial criterion for prohibition was the military potential of research projects rather than the difference between fundamental and applied research.

Similar to the American reaction to Bush’s proposals, discussions within the German scientific community over the dissociation of basic from applied research were quite controversial in the initial post-war years. Those from engineering or the applied sciences were particularly confused by this distinction and felt insecure about their future position and status within academia (Vieweg 1950: 731–732; Sörensen 1952: 158). The creation of compounds such as “applied fundamental research” (*angewandte Grundlagenforschung*) was a further German strategy designed to overcome this confusion in engineering (Heiss 1950: 121, 127; Wever 1952: 1053).

In order to cope with the Nazi past, the concept of pure science was initially more attractive because of its moral connotation in the sense of innocence. Many scientists labelled their research activities during the Nazi period retrospectively as pure science in order to avoid being accused of complying with and supporting the former fascist regime (Mehrtens 1994). In general, the revival of ideals belonging to the 19th-century concept of pure science was more extensive than in the US. Reference to the Humanist notion of education became part of the programme to democratise society. This notion thus shaped the self-understanding of German universities, which culminated in a re-glorification of the Prussian university reformer Wilhelm von Humboldt, who had emphasized the educational function of science.^19^ German professors embraced the older scientific ideal of truth-seeking as the ultimate motive for research.^20^

Right after the war, German academics tried hard to avoid the impression that their research was driven by any political or economic interests. The US occupying forces certainly wanted to keep science at a great distance to politics, but they did not seek to suppress economically and technically promising research (Cassidy 1996: 200–206). In fact, the growing tension with the Soviet Union meant that the Marshall Plan’s aim was speedy economic recovery in both Germany and Western Europe. John Krige has already pointed out that the basic-research concept played a key role in reconstructing European science under “American hegemony”. Firstly, the concept was important for communicating the US financial support for the former wartime enemies towards the American public. Secondly, it transported the Western ideology and was therefore part of the envisioned democratization process in central Europe. Finally, the US promoted basic research as unclassified research in the allied countries in order to increase its stock of scientific knowledge and thus to secure the American technological leadership (Krige 2010).

The German discourse on the general role of science in society defined scientific knowledge primarily as a cultural good in order to strip off the Nazi past: science was given a religious appeal (Walden 1946; Rein 1946; Reppe 1950: 1; Erbe 1954). However, even scientists such as the physicist Otto Hahn, who argued strongly that research in the natural sciences should abstain from any economic or technological considerations, advocating instead that science ought to be driven by the thirst for knowledge, campaigned for research funding by highlighting examples of scientific discoveries that eventually led to successful products or innovative technology (Hahn 1949, 1954).

Overall, the German concept of fundamental research resembled the US one in many ways. It encompassed the idea of the long-term perspective and that of the scientific knowledge reservoir or resource, the demand for scientific talent, individual creativity in research, and the belief in disciplinary specialisation (see, for instance, Reppe 1950). Eventually, fundamental research also became the key concept in the public funding of research in West Germany. However, the Germans’ attempt to institutionally dissociate academic research from research promising primarily economic utility was much more radical than in the US. West Germany founded the German Research Foundation in order to fund academic research and the Fraunhofer-Gesellschaft (Fraunhofer Society) as the funding body responsible for economically relevant research.

The German Research Foundation’s crucial criterion for defining fundamental research was the institutional autonomy of academic research. According to a number of historical studies, this particular focus on fundamental research implied a restoration of the power held by full professors (known in Germany as the *Ordinariensystem*). As the demands for the applied and the technical sciences were growing continually, in 1956, the German Research Foundation also established a special commission for funding applied research. However, this commission failed to gain importance as an instrument for promoting research in the technical sciences (Deutsche Forschungsgemeinschaft 1956). Technical universities therefore had to look for financial support from another quarter. In the end, the ideal of fundamental research in West Germany seemed to slow down the institutional emancipation of technical colleges from universities. Furthermore, academic research lost contact with expensive, major scientific projects carried out in publicly funded research institutes (Orth 2011).

All in all, the shift from a discourse of sustainability to one of purity after the Second World War appears to have been a transnational process, although both the background and the intensity of the purity ideals in the US and in West Germany differed from one another. In both nations, the purity discourse implied a revival of scientific ideals dating back to the 19th century. West Germany adopted the American imperative of basic research, but German scientists referred more extensively to the Humanist tradition of academia because they had to dissociate themselves from their Nazi past. As universities were supposed to play an important role in Germany’s effort to progress towards democracy, academic science was defined by primarily educational ideals. The call for basic research after the Second World War in the US initially sought to maintain federal funding for academic research in order to enable scientific talent to flourish without it being subject to pressure from the expectation of benefit held by society. Basic research became a key concept in US federal science policy because the latter defined science as a common good with a long-term perspective. However, the fact that this key concept became crucial in the long run can be explained only by the fact that it functioned as a discursive strategy designed to cope with the political and ethical dilemmas of science during the Cold War.

## Conclusions

This article has sought to demonstrate the importance of an historical approach in order to, firstly, understand the complex meanings of basic research and, secondly, answer the two questions of why science policy revolved around the concept of basic research and its dissociation from applied research for such a long time, and why this is still such a hot topic in science and technology studies today. If we continue to describe basic research as a timeless, clearly definable mode, even as an ideal type of research in contrast to applied research, we completely overlook the reason why this key concept in modern science policy emerged at all. In fact, this study has shown that the term basic research cannot be seen as a simple synonym for the older notion of pure science. As a consequence, the assumption made in social-scientific studies that the ideal of basic research structured modern science continuously up until the postmodern era, when application-oriented research was thought to gain predominance, needs to be corrected.

As the term basic research emerged in the early 20th century and became more common only in the late 1930s, it is actually quite young. Basic research is best described as a collective symbol of science policy designed to bridge the gap between the desire to support research, despite the fact that scientific output is unpredictable and that the expectations placed upon science by society have been growing constantly during the 20th century. For the history of basic research, it is crucial to note that the concept itself (as well as similar terms such as fundamental research) initially emerged in both the natural sciences within research fields that pursued explicitly practical ends and subdisciplines of engineering that targeted technological innovation and improvement.

While science profited financially from society’s growing demand for research, researchers simultaneously faced pressure from society’s expectation that science should produce immediately exploitable knowledge. In deploying the concept of basic research, scientists promised the public that research would lay the ultimate foundation for all sorts of progress and innovation, while at the same time conveying the experience that scientific research was time-consuming and its outcome and technical applications were hard to predict. Until 1945, basic research primarily meant long-term research in the natural sciences that was ultimately expected to solve practical problems.

After the Second World War, basic research became a central concept of US science policy, which particularly promoted research at universities and non-profit research institutes. Although the scientific promise of progress remained an important message in this concept after 1945, the discourse revolving around basic research shifted considerably in the post-war period from a discourse of knowledge sustainability to a discourse of purity. During the war, scientists had learnt to value massive governmental support of research, but they were concerned that the short-term planning of war-related research and its security restrictions would put the sustainability of both scientific knowledge and manpower at risk in the long run. Believing that scientists knew best when it came down to making science flourish and knowing what it took to explore the unknown, the challenge for scientists was legitimizing the continuance of federal science funding while at the same time advocating the institutional autonomy of science.

To this end, science policy advisers such as Vannevar Bush revived a long-lasting semantic reservoir of scientific ideals. By dissociating scientific knowledge from its potential applications, it became possible to define academic research as a common good capable of laying claim to federal protection, just as the older concept of pure science had done before. Bush’s proposal focused on the natural sciences, whose studies in the fundamental principles of nature were thought to offer nearly endless possibilities for technical innovation. Moreover, this new definition responded to the educational tasks undertaken by universities in which research projects were part of scientific qualification. The self-concept of higher education institutions traditionally kept their distance from any utilitarian aspects of scientific knowledge.

The distinction between basic and applied research thus served, first and foremost, as a criterion governing the allocation of federal funding, implemented through the newly founded National Science Foundation. Although the majority of researchers were grateful for the new federal support for research, the concept of basic research became the subject of controversies in the late 1940s because it reanimated ideals and norms of the older, European discourse of pure science. With these semantic references, basic research evoked older epistemic and social hierarchies. Research was seen to be more theory- than problem-oriented, the natural sciences assumed moral superiority over the technical sciences, and academic researchers were considered morally superior to industrial researchers. The individual pursuit of knowledge ennobled academic researchers, who became detached from immediate demands so that scientific creativity was given free rein.

To a certain extent, the re-establishment of older scientific ideals was a reaction to the exceptional conditions of wartime research. In many research fields, however, the ideals belonging to a former notion of science contradicted the changed practices in and demands placed on research in the 20th century. In particular, the idea of keeping technology apart from the natural sciences, which derived from an artificial funding demarcation, appeared anachronistic. These social and epistemic attributions of basic research looked like a cultural lag in modern science.

The reason why the concept of basic research, with all its reminiscences to former purity discourses, finally prevailed was that it functioned as a discursive strategy to cope with the difficult relationship between science and the public, the ideologically charged atmosphere of the Cold War, and the ethical dilemmas in science during the second half of the 20th century. When it comes to the political dimension of the concept of basic research, there are usually references to Robert Merton or Michael Polanyi, who stated that only democracy guaranteed full scientific autonomy and that, vice versa, scientific independence was a prerequisite of democratic pluralism because it presented a disinterested authority of truth (Merton 1942; Polanyi 1962). This self-image of science as being autonomous and disinterested was partly a result of Western ideology competing with the Soviet Union during the Cold War.

The effects of the Cold War on domestic politics were a major challenge to the scientific community. The first use of the atomic bomb rendered discussion of the goals of science unavoidable. Scientists who took part in the debate about the application of scientific knowledge for good or bad discovered that there was little room for negotiation in Cold-War America. At the same time, the relationship between the scientific community and the public became ever tenser as cooperation between science and the military increased. American intellectuals perceived this military-science nexus as a threat to US democratic culture. As a consequence, resorting to basic research was part of a strategy of relief – not only relief from society’s expectation of science to produce immediate benefits, but also from political controversies that might affect a researcher’s reputation and put his or her chances of acquiring funding at risk.

The success of this key concept in science policy lay in the polysemy of “basic”, which functioned as a kind of self-reassurance within the scientific community and could be used to signal societal utility when communicating to the wider public. The concept of basic research thus worked as collective symbol linking the public discourse to the scientific discourse. The label “basic” signified that research was a precondition for future scientific progress. At the same time, it communicated the fundamental importance of research for societal or economic and technological progress.

In the end, the concept of basic research could not solve the dilemmas of science in 20th-century societies. In fact, it produced its own confusion and misleading expectations. The simplified promise of progress depended on society’s confidence, which dwindled during the economic crisis of the 1970s. Particularly after the discourse on basic research referred to the intrinsic ideal of science for its own sake, society’s trust was put at risk. In addition, the simplified promise of scientific objectivity – the alleged neutrality of facts – obscured the actual complexity of research, where scientific truth is always contested.

Which lessons can we draw from this analysis for the current theoretical debate in science and technology studies? Bruno Latour’s argument against basic research is well taken in so far as the semantics of the concept do not represent the actual research practices and their institutional settings. As the above analysis has shown, the same type of criticism was already voiced by contemporaries of Vannevar Bush. Bush’s definition of basic research, especially his dissociation of the natural sciences from the technical sciences and its purity ideals, however, were more than just a simple misrepresentation: they had a long-lasting effect on the Western notion of science and research policy.

The resort to purity ideals can be blamed for retarding or interrupting this reflective process within the natural sciences. Moreover, there are hints that the reference to older scientific ideals led to taboos being placed on research topics leading to technical innovations, at least in some disciplines within the natural sciences. Eric J. Vettel has demonstrated how the revival of the policy of pure science in the 1950s altered research topics and institutional organization in microbiology and how the turn toward an application-oriented research policy during the 1960s led, in the long run, to biotechnology (Vettel 2006). The discipline of biology is thus a good example for demonstrating that parts of this purity discourse have, once again, faded away. The self-image of researchers like Craig Venter, geneticist and entrepreneur, no longer corresponds to the old image of the quiet academic scholar (Venter 2007). These recent historical shifts have indeed been noted by science studies. But we need more long-term historical studies on individual research fields and disciplines – studies tracing the historical development of research topics as far back as the late 19th and early 20th centuries – in order to understand the effects of the return of the purity discourse after 1945.

Bruno Latour has described purity discourses as a typically *modern* phenomenon that has since become less important. The results of this analysis, however, show that the concept of basic research had many functions. Aside from its role as a criterion for distributing research funding, the concept of basic research mainly served as a strategy for coping, firstly, with society’s increasing expectations of science, secondly, with the ethical dilemmas associated with the debate on the overall purpose of science, and, thirdly, with the political implications of science’s role as an increasingly powerful force in society. The case of the US shows that, despite the initial criticism of anachronism, the revival of the purity discourse succeeded because the concept of basic research became a strategy to cope with the uncertainties and dilemmas of the Cold-War period.

In fact, many of these dilemmas will continue to challenge science policy in the 21st century. As science and technology have become powerful forces in our societies, they will be subject to both conflicts of interest and political and ethical controversies. Whether the strategy of dealing with these controversies will continue to characterize basic research is, however, an open question. On the one hand, some representatives of science and technology studies believe that previous strategies have failed in coping with these dilemmas (Jasanoff 2005: 6; Shapin 2010: 387–391). On the other hand, new concepts like the European Research Council’s “frontier research” seem to revive the basic-research concept – at least with regard to its original function as part of a discourse on knowledge sustainability, which the current article has sought to reveal (High-Level Expert Group 2005: 16).^21^

Analysis of these discourses provides us with insights into expectations placed upon future research and into societal and scientific experiences from the past, which, among other things, frame decisions about what kind of research society wants and what kind of research should be funded by the public purse. Historical semantics can help to elucidate scientific taboos, which are taken for granted because they are the outcome of specific political or societal situations. All in all, historical semantics could be one useful approach among many in science and technology studies. It provides a critical perspective on the complex relationship between science and society. Moreover, it helps to reveal the historical legacy of our notions of science and technology, including their multiple attributes, which are still very present, although this seems to have gone unnoticed by many contemporary observers. This is why the analysis of concepts such as basis research is (still) an interesting, worthwhile subject for science studies. However, with regard to the legacy of the concept of basic research, this article suggests that it should not be used as a technical term whose meanings can be taken for granted. Scholars in the field of science and technology studies are thus well advised to explicate which of the many facets of the term they allude to when using the concept of basic research.

## Footnotes

1 See the subsection “The discursive strategy of ‘fundamental research’ and the reassessment of German science in the Nazi period” below for more details.

2 Given the recently growing interest in the relationship between science and technology, the reflection on terms like “applied science” seems to have started a little bit earlier (Bud 2012a, b; Gooday 2012; Karns Alexander 2012; Lucier 2012; Schatzberg 2012). This literature, however, focusses primarily on the 19th century.

3 In contrast to older approaches of historical semantics like intellectual history or the history of ideas, which cling to the idea of authorship and reception theory, discourse analysis does not aim at identifying the pedigree of new concepts or arguments in detail. Instead of singling out individual statements or discussing personal rationales, discourse analysis rather tries to find common patters of arguments that are shared by many discourse participants.

4 The cited sources and documents do not represent the whole sample of texts. The texts I refer to in this article were chosen because they exemplify broader discursive strategies revolving around the concept of basic research in a particularly typical way.

5 In the German-speaking context, the term science is not restricted to natural sciences. This also applied to the English-speaking academia in early modern times.

6 The history of both scientific organizations was the subject of two major research projects, which have recently been concluded. See the wealth of research published in the academic series *Beiträge zur Geschichte der deutschen Forschungsgemeinschaft* (Stuttgart: Franz Steiner Verlag, four volumes published so far) and *Geschichte der Kaiser-Wilhelm-Gesellschaft im Nationalsozialismus* (Göttingen: Wallstein Verlag, seventeen volumes published so far).

7 In this article, I will consistently translate the German term *Grundlagenforschung* as “fundamental research”. Unlike the Anglo-American science discourse, the German science discourse does not accommodate conceptual variations such as that between “basic research” and “fundamental research”.

8 Trained as a theologian, Hans Hartmann worked as an author specializing in writing about scientists and science policy issues.

9 In other disciplinary contexts, the study of material properties was also denoted as goal-oriented research (Bauermeister 1938: 479).

10 In the natural sciences during this period, the term only appeared in a very specific context. In the 1920s, a group of biologists and psychologists discussed the question of the functional usefulness of organs or mental processes within organisms, an approach they also labelled as *Zweckforschung* (E. Becher 1921: 296–304; H. Becher 1923).

11 It took a while before the new term *Zweckforschung* (goal-oriented research) was established. Confusingly, some Nazi partisans used the term goal-oriented research as a synonym for pure science, criticizing the idea of research as an end in itself (W. Schultze 1938: 5; Löhr 1938: 20).

12 In order to get a better idea of conceptual dissemination, I provide here some results of a statistical analysis of the electronically accessible journal *Science* (including the supplement *The Scientific Monthly*). Between 1921 and 1930, the term basic research was used 14 times and fundamental research was used 121 times. Between 1931 and 1940, the term basic research showed up 51 times and fundamental research 154 times. Just five years later, the term basic research had been employed 69 times and fundamental research 182 times.

13 See, for instance, the decision of the Senate of the Ludwig Maximilian University in Munich in the early 1920s (Archive of the University, Sen. 523).

14 Bush consistently referred to “basic research” in his report. At the time, however, the term “fundamental research” was more widespread.

15 As the broader research context was hidden to scientific peers and to the researchers themselves, this strategy at least allowed the publication of detailed results.

16 In 1944 and 1945 there was already an ongoing debate in letters to the editor of the journal *Science* that critically reflected the re-idealization of the notion of pure science (Stern 1944; Pearson 1944; Robin 1944; Feibleman 1944; Alexander 1945; Stern 1945; Moore 1945; J. R. Baker 1945).

17 See also James C. Connant’s contribution to the debate on the relationship between science and the public (1951). For more details, see Hershberg (1993: 554–577).

18 The exact German terms deployed in the two key laws, the Kontrollratsgesetz no. 25 from 29.04.1946 and the Militärregierungsgesetz, no. 23 from 12.09.1949 were “grundlegende wissenschaftliche Forschung” and “angewandte wissenschaftliche Forschung”. The regulations defined military relevance on the basis of several classified research fields. For more details of how the allies controlled research, see Heinemann (2001).

19 In the German university tradition, Humboldt represents the link between education and research. For the current state of research on Humboldt’s role in university discourse, see Eichler (2012), Jarausch (1999).

20 The semantic differences between the two versions of the speech held by philosopher Karl Jaspers in 1923 and 1946 are significant when defining the role of universities in the wake of changes to the respective political systems: while Jaspers emphasized the objectivity of scientific knowledge in 1923, he replaced the term “objectivity” with “truth” in 1946 (Jaspers 1923; 1946).

21 The definition of frontier research relies again on the basic-applied nomenclature. In contrast to recent trends of promoting more applied forms of research, it shifts the balance further towards the basic-research pole of the continuum. See also the mission statement on the ERC website: http://erc.europa.eu/mission.
